# Structures and dynamics of the major G-quadruplex in the human PDGFR-β gene promoter: insights into vacancy G-quadruplex formation

**DOI:** 10.1093/nar/gkag843

**Published:** 2026-09-04

**Authors:** Yichen Han, Jonathan Dickerhoff, Danzhou Yang

**Affiliations:** Borch Department of Medicinal Chemistry and Molecular Pharmacology, College of Pharmacy, Purdue University, West Lafayette, IN 47906, United States; Borch Department of Medicinal Chemistry and Molecular Pharmacology, College of Pharmacy, Purdue University, West Lafayette, IN 47906, United States; Borch Department of Medicinal Chemistry and Molecular Pharmacology, College of Pharmacy, Purdue University, West Lafayette, IN 47906, United States; Purdue Institute for Cancer Research, James Tarpo Jr. and Margaret Tarpo Department of Chemistry, Purdue Institute for Drug Discovery, Purdue University, West Lafayette, IN 47906, United States

## Abstract

Overexpression of PDGFR-β (platelet-derived growth factor receptor beta) kinase contributes to diverse human diseases, including cancers, cardiovascular disorders, and fibrosis. G-quadruplexes (G4s) formed in the PDGFR-β promoter act as transcriptional repressors and represent attractive therapeutic targets. We previously reported that the major G4-forming region of the PDGFR-β promoter adopts a unique broken-strand G4, whereas truncation of this sequence generates a vacancy G4 (vG4) that can be filled-in by external guanine analogs or metabolites and further stabilized by small molecules, suggesting a potential regulatory mechanism and opportunity for selective drug targeting. However, the relationship between broken-strand G4s and vG4s remains unclear. Here, we demonstrate that the PDGFR-β promoter sequence forms a dynamic equilibrium between two broken-strand G4 conformations that interconvert on the millisecond timescale, with vG4 serving as an intermediate. We determined the high-resolution NMR structures of these interconverting G4s, which share a conserved vG4 core but differ in their intramolecular guanine “fill-in.” Both conformations feature a stabilizing G–G capping base pair unique to the PDGFR-β promoter. These findings elucidate the structural details of broken-strand PDGFR-β promoter G4s and the mechanism of vG4 formation, providing critical insights for selective drug targeting and establishing a framework for rational design of small molecules to modulate PDGFR-β transcription.

## Graphical Abstract

**Graphical Abstract:**
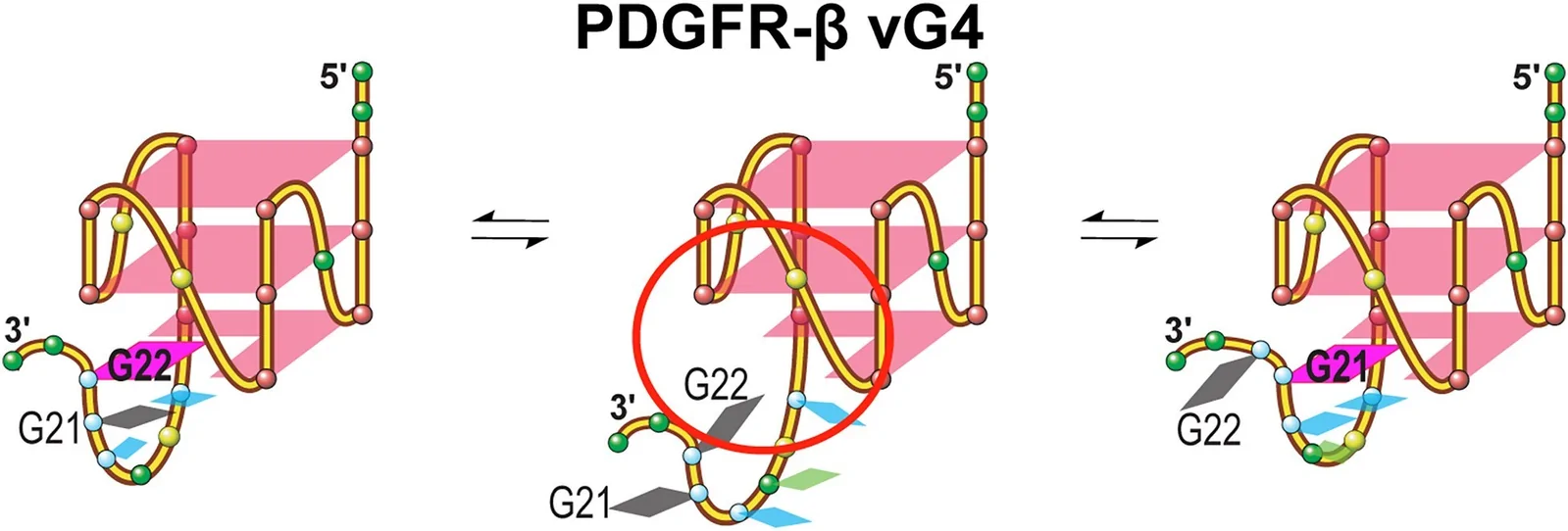

## Introduction

The platelet-derived growth factor receptor β (PDGFR-β) protein is a cell-surface receptor tyrosine kinase that plays a critical role in regulating cell proliferation, survival, motility, and differentiation [1, 2]. Aberrant PDGFR-β overexpression contributes to the development of multiple human diseases, including various cancers—where it promotes tumor growth and metastasis—as well as cardiovascular disorders [1–5]. Therefore, PDGFR-β represents an attractive therapeutic target, and several kinase inhibitors have been evaluated in clinical trials [1, 2, 6]. However, these inhibitors often lack specificity [6].

DNA G-quadruplexes (G4s) are four-stranded secondary structures that form in guanine-rich sequences [7]. They are particularly enriched in oncogene promoters, where they play important regulatory roles in transcription, and have emerged as an attractive class of cancer-specific molecular targets [8–10]. The building block of a G4 is the planar G-tetrad, in which four guanine bases are connected through Hoogsteen hydrogen bonds [11]. Stacking of two or more G-tetrads forms the G4 core, which is critically stabilized by coordination of monovalent cations, particularly potassium ions (K^+^) [11].

**Figure 1.**
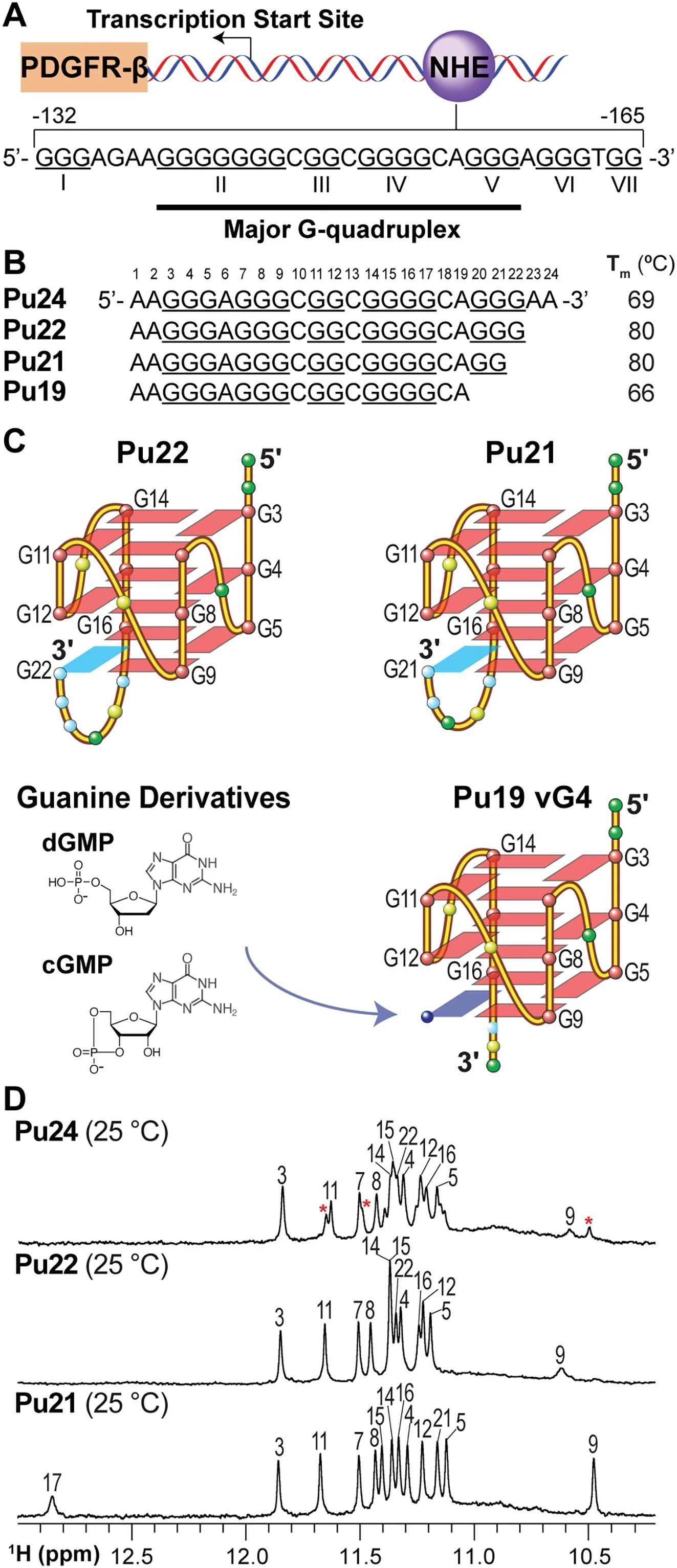
**A**) G-rich NHE sequence of the human PDGFR-β promoter, with the major G4 region underlined. (**B**) Pu24, Pu22, Pu21, and Pu19 sequences with melting temperatures (*T*_m_ in 100 mM K^+^). (**C**) Folding of the Pu22, Pu21, and Pu19 (vG4) G-quadruplexes. Pu19 vG4 can be filled-in by guanine derivatives. (**D**) 1D ^1^H NMR spectra showing imino regions of Pu24, Pu22, and Pu21 at 25°C. Asterisks: Pu24 minor species peaks. The imino proton assignment of Pu22 was based on a previous ^15^N-labeling experiment [15]. NMR sample: 150 μM DNA, 100 mM K^+^, pH 7, 10% D_2_O/90% H_2_O.

The proximal promoter of the human PDGFR-β gene has a GC-rich nuclease-hypersensitive element (NHE) that forms DNA G4, which functions as transcription repressor, silences PDGFR-β expression upon stabilization by small molecules, and represents a promising target for transcriptional modulation (Fig. 1A) [12–16]. The major G4 forming region of the PDGFR-β promoter NHE comprises G-runs II–V consisting of seven, two, four, and three guanines, respectively (Fig. 1A) [15]. We previously determined the folding topology of the major PDGFR-β G4 using the Pu22 sequence, which adopts a three-tetrad parallel structure with a novel broken-strand motif in physiological K^+^ solution (Fig. 1B and C, Pu22) [15]. Unexpectedly, rather than utilizing continuous three-guanine tracks from G-runs II, IV, and V, the structure incorporates 2G-run III (G11 and G12), which is completed with the 3′-terminal G22 from G-run V to form a broken G-strand in the G-tetrad core. This unique folding pattern maximizes the number of 1-nt chain-reversal loops, resulting in three such loops within a three-tetrad parallel G4 [11, 15–25].

Upon further investigation, we found that truncating the fill-in G-run V of the PDGFR-β promoter sequence forms a vacancy G-quadruplex (vG4) with an incomplete G-tetrad (Fig. 1B and C, Pu19) [27]. This vG4 can be filled-in by external guanine derivatives such as cGMP or GTP to complete the vacancy G-tetrad (Supplementary Fig. S1), which can be further stabilized by small molecule targeting [28, 29]. Recent studies have identified potential vG4-forming motifs widely distributed in gene promoter regions [30, 31].

Intriguingly, the broken-strand G4 formed by the more extended Pu22 sequence can be viewed as an intramolecular fill-in vG4, where the 3′-terminal G22 from G-run V fills the vacancy (Fig. 1C, Pu22). However, the intramolecular fill-in by G22 appears to preclude vG4 formation. Therefore, whether and how vG4 can form in the extended PDGFR-β promoter remains unclear.

In this study, we report that the extended PDGFR-*β* promoter sequence forms a mixture of two broken-strand G4s in K⁺ solution (Fig. 1C, Pu22 and Pu21). We determined the high-resolution nuclear magnetic resonance (NMR) structures of these two interconverting G4s. Both structures share a parallel-stranded G4 core with a vacancy in the 3′ G-tetrad, which is filled by either G22 or G21 from G-run V via a snap-back loop. This snap-back loop forms a unique hairpin structure with a reverse-Hoogsteen G–G base pair. NMR analysis revealed that these two broken-strand G4s interconvert in the slow-exchange regime on the NMR timescale, with the G22-filled structure being the predominant conformation. The interconversion of the two broken-strand structures requires a vG4 intermediate, which can allow external guanine metabolite fill-in. These findings establish the broken-strand G4s and vG4 structures in the PDGFR-β promoter as unique and druggable elements, and the structural insights provide a critical foundation for rational drug design targeting PDGFR-β transcriptional regulation.

## Materials and methods

### Sample preparation

All oligonucleotides were synthesized and purified as previously described, with additional ethanol precipitation for 2D NMR samples [15]. NMR samples were made by dissolving lyophilized DNA powder in 10–100 mM K-phosphate/KCl, pH 7.0, buffer containing 10% D_2_O/90% H_2_O. NMR samples were annealed at 95°C for 5 min and cooled slowly at room temperature. DNA concentration was quantified based on their UV/Vis absorbance at 260 nm.

### Nuclear magnetic resonance experiments

NMR spectra were measured on a Bruker Avance-III 800 MHz spectrometer with a QCI cryoprobe. NOESY spectra were collected with mixing times of 50–400 ms at temperatures of 5°C–40°C. EASY-ROESY spectrum was collected with a mixing time of 80 ms and a tilt angle of 43° [32]. ^1^H-^13^C HSQC spectra were collected at 25°C and 40°C with a coupling constant of 145 Hz. Topspin 3.6.2 (Bruker) and CcpNmr 2.4.2 (CCPN) were used to process and assign NMR spectra [33].

To determine the exchange rate for the interconversion between Pu22-in and Pu21-in in the Pu24 sequence, a series of NOESY spectra were collected with increasing mixing times (50, 150, 250, 400 ms) at 25°C. The A23H8 exchange cross peak was integrated and normalized against its own diagonal peak at each mixing time. Exchange rate (*k*_ex_) was determined by fitting the A23H8 exchange peak buildup versus mixing time based on the Bloch–McConnell model [34]:


\begin{eqnarray*}
{{I}_{\mathrm{normalized}}} = \frac{{1 - \mathrm{ EXP}\ \left( {{{k}_{\mathrm{ ex}}}\times{{\tau }_\mathrm{ m}}} \right)}}{{1 + \mathrm{ EXP}\ \left( {{{k}_{\mathrm{ ex}}}\times{{\tau }_\mathrm{ m}}} \right)}}.
\end{eqnarray*}

### Circular dichroism experiments

Circular dichroism (CD) experiments were performed on a JASCO-1100 spectropolarimeter with a temperature controller (JASCO, Inc). DNA samples were prepared in 25 mM K-phosphate/75 mM KCl, pH 7.0, buffer at a concentration of 5 μM. CD spectra were collected through a quartz cell with 1 cm path length, 1 nm bandwidth, and 1 s response time at 25°C. CD spectra were blanked by subtracting a buffer spectrum. Melting curves were collected by recording the 264 nm CD signal over a temperature range of 25°C–95°C at a ramp rate of 1°C/min.

### Structure calculation

Hydrogen bond, planarity, dihedral, and NOE-based distance restraints were assigned following the previously described scheme [35]. Xplor-NIH and Amber structure calculation was conducted using the previously described procedure [35, 36, 37]. Top 10 lowest-energy structures were used for Protein Data Bank deposition.

## Results and discussions

### The extended PDGFR-β promoter G4-forming sequence forms two equilibrating broken-strand structures, Pu22 and Pu21

Using the Pu22 sequence from the major G4-forming region of the PDGFR-β promoter, we previously showed that it adopts a broken-strand parallel G4 structure with an intramolecular fill-in from the 3′-terminal G22 (Fig. 1C, Pu22) [15]. To investigate the structure and dynamics of this G4 with a non-terminal G22 as in the actual promoter context, we extended Pu22 at the 3′ end by two adenosine residues to generate the Pu24 sequence (Fig. 1B, Pu24). We recorded the 1D ^1^H NMR spectra of Pu24 in K^+^ solution at 5°C and 25°C (Fig. 1D and Supplementary Fig. S2A and B, Pu24). The 10–12 ppm region of the 1D ^1^H NMR spectrum corresponds to G-tetrad imino protons, with 1 peak per guanine and 12 peaks expected for a three-tetrad G4. At both temperatures, the Pu24 spectra exhibited >12 imino proton peaks, indicating the presence of multiple G4 species (Fig. 1D and Supplementary Fig. S2B). Very similar spectra (Supplementary Fig. S2C) were also obtained for Pu24 in the presence of Na^+^ cations at intracellular concentration (5 mM) and close to human body temperature (35°C).

In the Pu22 broken-strand G4, the imino proton of G9—directly hydrogen-bonded to the fill-in G22—is most upfield-shifted and isolated from other imino peaks (Fig. 1D, Pu22). Notably, the Pu24 spectra show two clear peaks for the G9 imino proton, with one at the same location as Pu22 G9, indicating two co-existing species including the Pu22 G4. Comparative analysis of the Pu24 and Pu22 spectra (Fig. 1D) reveals that the imino peaks of Pu22 closely match the major subset of peaks in Pu24, strongly suggesting that the Pu22 G4 represents the major species formed by Pu24.

As G9 in the 3′ external G-tetrad is hydrogen-bonded with the fill-in G22 (Fig. 1C), the two similarly upfield-shifted G9 imino signals observed in Pu24 suggest that both equilibrium species adopt a comparable fill-in conformation. Furthermore, the imino proton peaks of the minor species in Pu24 closely resemble those in Pu22 (Fig. 1D), providing additional support for a similar structure.

Sequence analysis of Pu24 suggests that G21 or G20, located within the same G-run (V) as G22, may serve as alternative fill-in guanines (Fig. 1B). To test the potential involvement of the adjacent G21, we truncated Pu22 to generate the Pu21 sequence (Fig. 1B, Pu21). The 1D ^1^H NMR spectrum of Pu21 revealed 12 sharp imino proton peaks, indicative of a well-defined three-tetrad G4 (Fig. 1D, Pu21). Notably, the imino peaks of Pu21 closely resemble the minor peaks observed in Pu24, with the most upfield-shifted G9 imino peak in Pu21 matching the G9 minor peak in Pu24. This strongly suggests that the Pu21 G4 corresponds to the minor species formed in Pu24. This interpretation is further supported by previous DMS footprinting of the extended PDGFR-β sequence, which showed partial protection of G21, consistent with its involvement in G-tetrad formation [15]. In contrast, the Pu20 sequence with truncation of both G22 and G21 (Supplementary Table S1) did not show the formation of a well-defined G4 in its 1D ^1^H NMR spectrum (Supplementary Fig. S2D, Pu20).

Taken together, these results indicate that the extended PDGFR-β promoter sequence Pu24 adopts two interconverting G4 conformations with either a G22 or G21 fill-in, corresponding to those formed by the Pu22 and Pu21 sequences, respectively (Fig. 1C).

### Pu22 and Pu21 form three-tetrad parallel G-quadruplexes with a broken strand as shown by NMR

Both Pu22 and Pu21 PDGFR-β sequences exhibit excellent NMR spectral quality and are well-suited for high-resolution structural determination. They both show 12 sharp imino proton peaks, indicating a well-defined three-tetrad G4 (Fig. 1D).

To gain structural insights into the two interconverting G4s, we determined their high-resolution NMR structures in K^+^ solution. We acquired 2D ^1^H-^1^H NOESY (Nuclear Overhauser Effect Spectroscopy) and ^1^H-^13^C HSQC (Heteronuclear Single Quantum Coherence) spectra for Pu22 and Pu21 at various temperatures (5°C–40°C) and K^+^ concentrations (10–100 mM). Complete proton resonance assignment was achieved based on NOESY, HSQC, and previous ^15^N site-specific labeling experiments of the Pu22 G4 (Supplementary Tables S2–S5) [15, 38].

**Figure 2.**
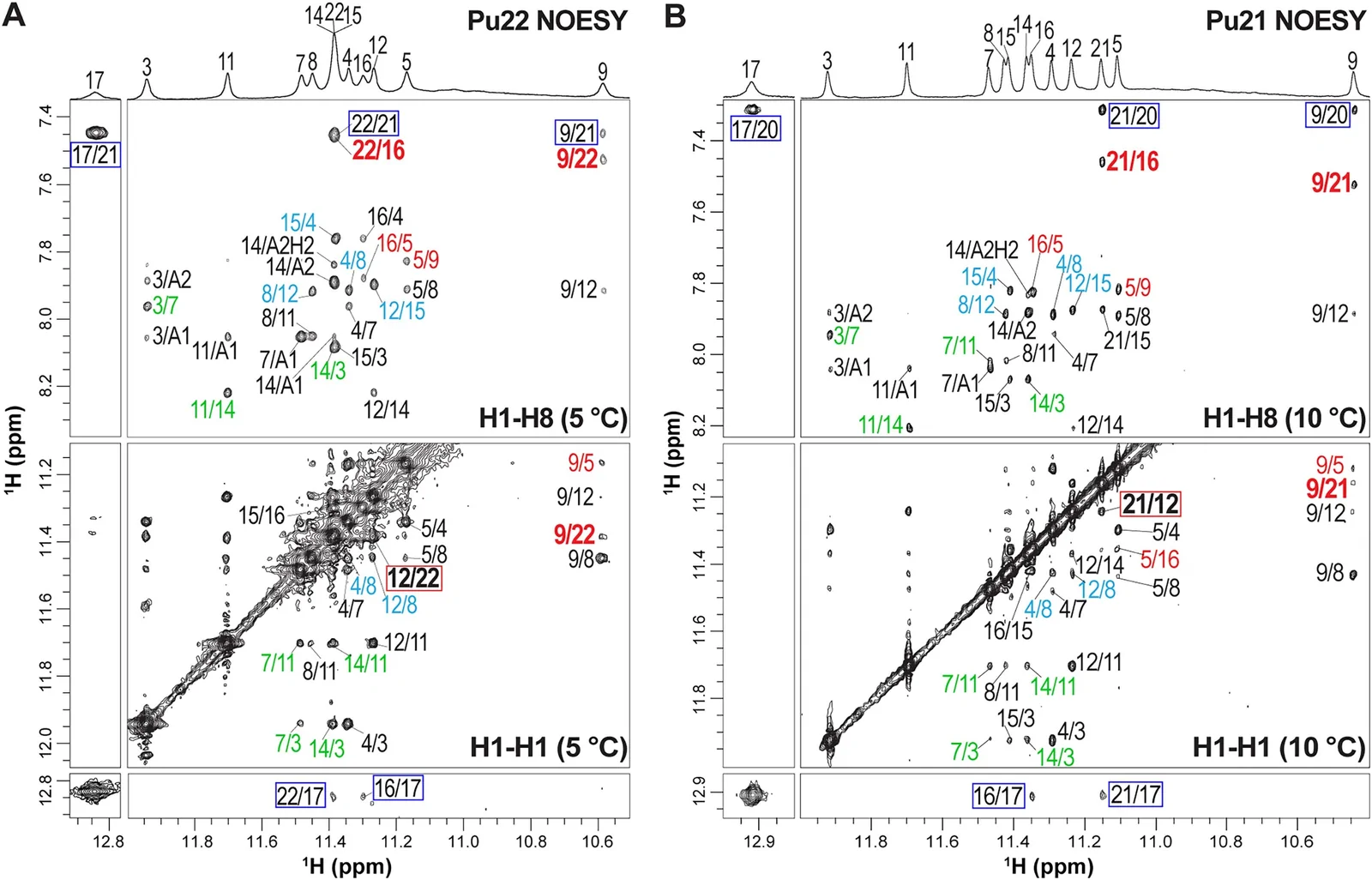
2D NOESY H1–H8 and H1–H1 spectral regions of the (**A**) Pu22 (5°C) and (**B**) Pu21 (10°C) G4s. Red label = intra-tetrad NOEs defining the fill-in 3′-tetrad. Red boxes = H1–H1 NOEs showing the G22/21 fill-in. Cyan label = intra-tetrad NOEs of the middle tetrad. Green label = intra-tetrad NOEs of the 5′-tetrad. Blue box = NOEs defining the 3′-capping G17:G21/20 base pair. NOESY were measured with a mixing time of 250 ms. NMR sample: 1 mM DNA, 100 mM K^+^, pH 7, 10% D_2_O/90% H_2_O.

Within each G-tetrad, H1 guanine imino protons exhibit NOEs with neighboring H1 and H8 protons, defining the tetrad core (Fig. 2). For both Pu22 and Pu21 G4s, intra-tetrad H1–H1 and H1–H8 NOEs delineate the 5′-tetrad (G3–G7–G11–G14) and central tetrad (G4–G8–G12–G15) (Fig. 2). Inter-tetrad NOEs—G3H8–G15H1, G7H8–G4H1, G11H8–G8H1, and G14H8–G12H1—connect the 5′ and central tetrads, indicating a right-handed twist of the DNA backbone (Fig. 2). Sequential NOEs among continuous G-strands (e.g. G3–G4–G5, G7–G8–G9, and G14–G15–G16) further support the strand topology (Fig. 2 and Supplementary Figs S3 and S4).

In Pu22, the 3′-terminal tetrad comprises G5–G9–G22 (fill-in)–G16, with G22 acting as a non-adjacent fill-in guanine from the 3′-end (Fig. 1C). This is supported by intra-tetrad NOEs including G22H1–G16H8, G22H1–G9H1, G9H1–G22H8, and by the inter-tetrad NOEs G22H1–G12H1 and G22H8–G12H8 (Fig 2A and Supplementary Fig. S3). Similarly, Pu21 exhibits analogous NOEs for its 3′-terminal G21 residue (Fig 2B and Supplementary Fig. S4), confirming G5–G9–G21 (fill-in)–G16 tetrad (Fig. 1C).

Furthermore, H8–H8 and H1–H1 NOEs (Fig. 2 and Supplementary Figs S3 and S4) are observed along the broken strand of both Pu22 and Pu21, including those of G11–G12, as well as G22/G21–G12 H8–H8, and H1–H1 NOEs, confirming the G22/G21 fill-in for Pu22 and Pu21, respectively. Therefore, these data demonstrate that Pu21 adopts the same vG4 core as Pu22, with the terminal G21 serving as the fill-in guanine (Fig. 1C). G4-strands of both Pu22 and Pu21 have the same direction and are connected by the three 1-nt double-strand reversal loops of A6, C10, and C13, as shown by their lack of sequential NOE contacts with adjacent guanines (Supplementary Figs S5 and S6). The parallel-stranded G4s are supported by CD spectra, which show the characteristic negative 240 nm and positive 265 nm signatures (Supplementary Fig. S7). Thus, Pu22 and Pu21 both form three-tetrad, broken-strand parallel G4s, with G22 or G21 fill-in at the 3′-tetrad.

### 3′-Terminal fill-in of G22 or G21 via distinct snap-back loops with conserved G:G capping

With core G-tetrad guanine protons assigned, all NOEs of Pu22 and Pu21 were subsequently assigned (Supplementary Figs S8 and S9). Confirming the G-tetrad core topology, sequential NOEs between aromatic H8 and sugar H1′/2′,′′/3′ protons were observed within each continuous G-core strand, as well as for the broken-strand fill-in G22/G21H8, which has clear sequential NOEs with G12 sugar proton H1′/2′,′′/3′ (Supplementary Figs S5 and S6). Notably, all tetrad Gs, including the fill-in G22/G21, adopt an *anti* glycosidic conformation as shown by NOESY (Supplementary Figs S5 and S6) and ^1^H-^13^C HSQC (Supplementary Fig. S10) [39].

For the Pu22 and Pu21 G4s, the fill-in G22 and G21 are delivered by a 5-nt or 4-nt snap-back loop, respectively (Fig. 1C). G17 stacks over G16 and G22/G21 of the 3′-tetrad as shown by G17H8–G16H8, G17H1–G16H1, and G17H1–G22/G21H1 NOEs (Supplementary Figs S8 and S9), while G21 of Pu22 and G20 of Pu21 stack over the 3′ fill-in guanine (Fig. 1C).

Intriguingly, the exchangeable imino proton (H1) of the non-tetrad G17 is clearly observed in both Pu22 and Pu21 spectra (Fig. 1D and Supplementary Fig. S2B), suggesting its involvement in a stable hydrogen-bonded conformation. Notably, G17H1 exhibits distinct NOEs with Pu22/G21H8 or Pu21/G20H8 (Fig. 2). The downfield shifted G17H1 (Supplementary Fig. S2B) indicates a NH—N hydrogen-bonding instead of NH—O [40]. Additionally, G17H8 and H1, as well as Pu22/G21H8 or Pu21/G20H8, show NOEs with the imino protons of the 3′-tetrad guanine G16, G9, and the fill-in guanine G22 or G21 (Supplementary Figs S8 and S9). These observations indicate that G17 forms a reverse-Hoogsteen G:G base pair with the Pu22/G21 or Pu21/G20 adjacent to the 3′-terminal fill-in guanine, stacking over the 3′ G-tetrad.

The G17:G21/G20 capping base pair is connected by a 3-nt loop C18-A19-G20 in Pu22 and a 2-nt loop C18-A19 in Pu21. A19 of the 2-nt loop in Pu21 stacks well with the G17:G20 base pair as shown by NOEs between A19H8 and G17H8/G20H8 as well as NOEs between A19H2 and G17 sugar (Supplementary Fig. S9), whereas C18 is not well-stacked as evidenced by much weaker NOEs. In contrast, fewer and weaker inter-residue NOEs are observed for the 3-nt loop in Pu22 compared to Pu21 (Supplementary Fig. S8), indicating a more dynamic conformation. This is corroborated by the weaker Pu22 G17H1 NMR peak, which is only visible at 5°C in contrast to the Pu21 G17H1 that is even observable at 25°C, and the much broader Pu22 G9H1 resonance at 25°C (Fig. 1D and Supplementary Fig. S2B). Notably, G20 in Pu22 also stacks with the G17:G21 base pair as shown by G20H8–G21H8 NOE (Supplementary Fig. S3B). However, G20 adopts a *syn* conformation, as shown by NOESY (Supplementary Fig. S5) and HSQC (Supplementary Fig. S10A), in contrast to the *anti* A19 in Pu21 (Supplementary Fig. S10B). As a result, while Pu22 and Pu21 show very similar imino proton chemical shifts for the 5′ and middle G-tetrads, those of the 3′-tetrad, such as G22/G21 and G16, are clearly different (Fig. 1D), likely due to the distinct 3′ loop conformations.

The 5′-flanking of both Pu22 and Pu21 is identical and contains two adenosine residues A1 and A2, which show very similar NOEs with the 5′-tetrad, suggesting the same capping structures with the 5′-tetrad (Supplementary Figs S8 and S9). A1 stacks over G7, with A1H2 pointing toward the G7 tetrad edge, as supported by NOEs between A1H2 and G7H8/H1′, while A1H8 is positioned near the 5′-tetrad center as indicated by NOEs with all 5′-tetrad guanine imino protons (Supplementary Figs S8 and S9). A2 stacks over G14, as shown by A2H8/H2–G14H1 and A2H2–G14H1′ NOEs (Supplementary Figs S8 and S9). The A1 sugar locates above the G7–G3 edge, as indicated by NOEs with G3H1 and G7H8, while A2 sugar is above G3–G14 edge as shown by NOEs with G3H8 and G14H1 (Supplementary Figs S8 and S9). Notably, A1 adopts a *syn* conformation based on NOESY and HSQC (Supplementary Figs S5, S6, and S10) and appears to orient head-to-head with A2 base as shown by a NOE between A1H8 and A2H8 (Supplementary Figs S3 and S4).

Taken together, the 3′-terminal G22 in Pu22 and G21 in Pu21 serve as fill-in guanines via distinct snap-back loops, yet both structures share a conserved G:G base-pair capping motif.

### High-resolution NMR structures of the PDGFR-β Pu22 and Pu21 G4s

To determine the NMR structures of the Pu22 and Pu21 G4s, NOE-based distance restraints were derived and used for structure calculations (Supplementary Figs S8 and S9, Supplementary Tables S6–S9). A total of 755 and 710 NOE-based distance restraints were applied for Pu22 and Pu21, respectively, using restrained molecular dynamics. The final NMR ensembles consist of the 10 lowest-energy structures (Supplementary Figs S11 and S12), with overall RMSDs of 0.71 Å for Pu22 (PDB ID: 9P9X) and 0.61 Å for Pu21 (PDB ID: 9P9Y) (Supplementary Table S10).

**Figure 3.**
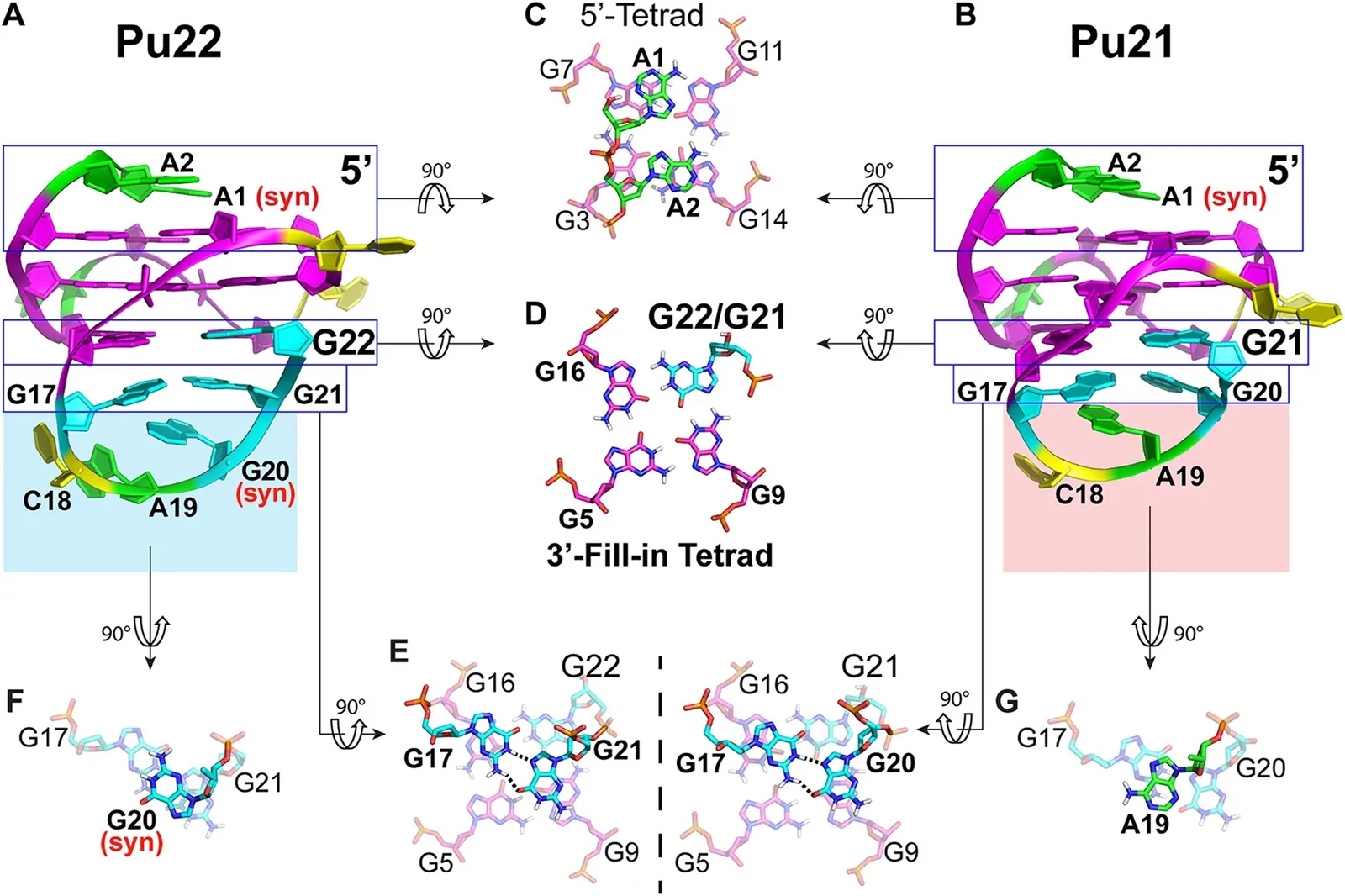
High-resolution NMR structures of the major PDGFR-β (**A**) Pu22 and (**B**) Pu21 G4s. (**C**) A1 and A2 stack on the 5′-tetrad. (**D**) 3′-tetrad structure showing the fill-in G. (**E**) G17:G21 and G17:G20 reverse Hoogsten base pairs stack on the 3′-tetrad in Pu22 and Pu21, respectively. (**F**) Loop G20 (*syn*) stacks on the G17:G21 base pair Pu22 G4. (**G**) Loop A19 (*anti*) stacks on the G17:G20 base pair in the Pu21 G4.

Both Pu22 and Pu21 adopt three-tetrad, broken-strand, right-handed parallel G4 structures (Fig. 3A and B). The 3′-terminal guanine (G22 in Pu22 and G21 in Pu21) serves as a fill-in residue, forming Hoogsteen hydrogen bonds with G16 and G9 to complete the 3′-tetrad (Fig. 3D). The four parallel G-strands are connected by three 1-nt double-reversal loops (A6, C10, and C13). The broken-strand folding appears to be driven by maximizing the number of 1-nt double-strand reversal loops that are highly favorable in a three-tetrad parallel-stranded G4 (Fig. 1C).

At the broken-strand junction, the fill-in guanine delivered by the snap-back loop has an opposite strand direction relative to the G11–G12 strand and typically adopts a *syn* conformation, as seen in other broken-strand G4s with snap-back loops [41–44]. Interestingly, in both Pu22 and Pu21, the fill-in guanines adopt an *anti* conformation. This results in a sugar-phosphate backbone orientation that matches the G11–G12 strand but is reversed relative to the preceding residue in the snap-back loop (Fig. 3A and B).

The 3′-terminal fill-in guanine is delivered via a 5-nt snap-back loop in Pu22 and a 4-nt loop in Pu21. Both loops contain a reverse-Hoogsteen G:G base pair—G17:G21 in Pu22 and G17:G20 in Pu21—capping the 3′-tetrad (Fig. 3E). The G:G base pair is connected by a loop turn: C18–A19–G20 in Pu22 and C18–A19 in Pu21, each adopting distinct conformations. In Pu22, G20 adopts a *syn* conformation and primarily stacks with G21 (Fig. 3F). In contrast, in Pu21, A19 stacks on both G17 and G20, with A19H8 located centrally and A19H2 near G17 (Fig. 3G). The 5-nt snap-back loop in Pu22 appears more dynamic, as evidenced by the broader G9H1 peak compared to Pu21 (Fig. 1D and Supplementary Fig. S2B). Despite the differences in loop conformation and dynamic, both Pu22 and Pu21 exhibit similar CD melting temperatures ∼80°C (Supplementary Fig. S7), suggesting comparable thermal stability.

The unique G:G base pair within the snap-back loop appears to stabilize the broken-strand G4. Mutating either G17 or G21/G20 to thymine notably reduces the melting temperature (Supplementary Fig. S13), while the G21/G20 mutation shows more disruptive effects. This explains why the Pu20 sequence fails to form a stable fill-in G4 and likely exists as a mixture of two vG4 conformers (Fig. 1D).

The 5′-capping structure is identical in both Pu22 and Pu21 G4s (Fig. 3C). Adenosines A1 and A2 stack over the 5′-tetrad: A1 stacks over G7 with its sugar positioned above the G7–G3 edge, and A2 stacks over G14 with its sugar above the G3–G14 edge. A1 and A2 adopt a head-to-head orientation, with A1 in a *syn* conformation forming an intra-residue N3–HO5′ hydrogen bond, a feature commonly observed for 5′-terminal purine residues [45].

### G22-fill-in and G21-fill-in G-quadruplexes interconvert on the millisecond timescale in the extended PDGFR-β promoter

The 1D ^1^H NMR spectrum of the extended PDGFR-β promoter sequence Pu24 reveals the coexistence of two equilibrating species: the Pu22 and Pu21 G4s (Fig. 1C). To investigate the dynamics of these structures within Pu24, we performed 2D NMR experiments at various temperatures. NOESY assignments indicate that the G22-fill-in G4 is the major species (Supplementary Figs S14 and S15). Notably, the non-terminal filled-in G22 in Pu24 still adopts an *anti* glycosidic conformation, as confirmed by both NOESY and HSQC spectra, indicating that its conformational preference is retained despite its non-terminal position (Supplementary Fig. S16).

**Figure 4.**
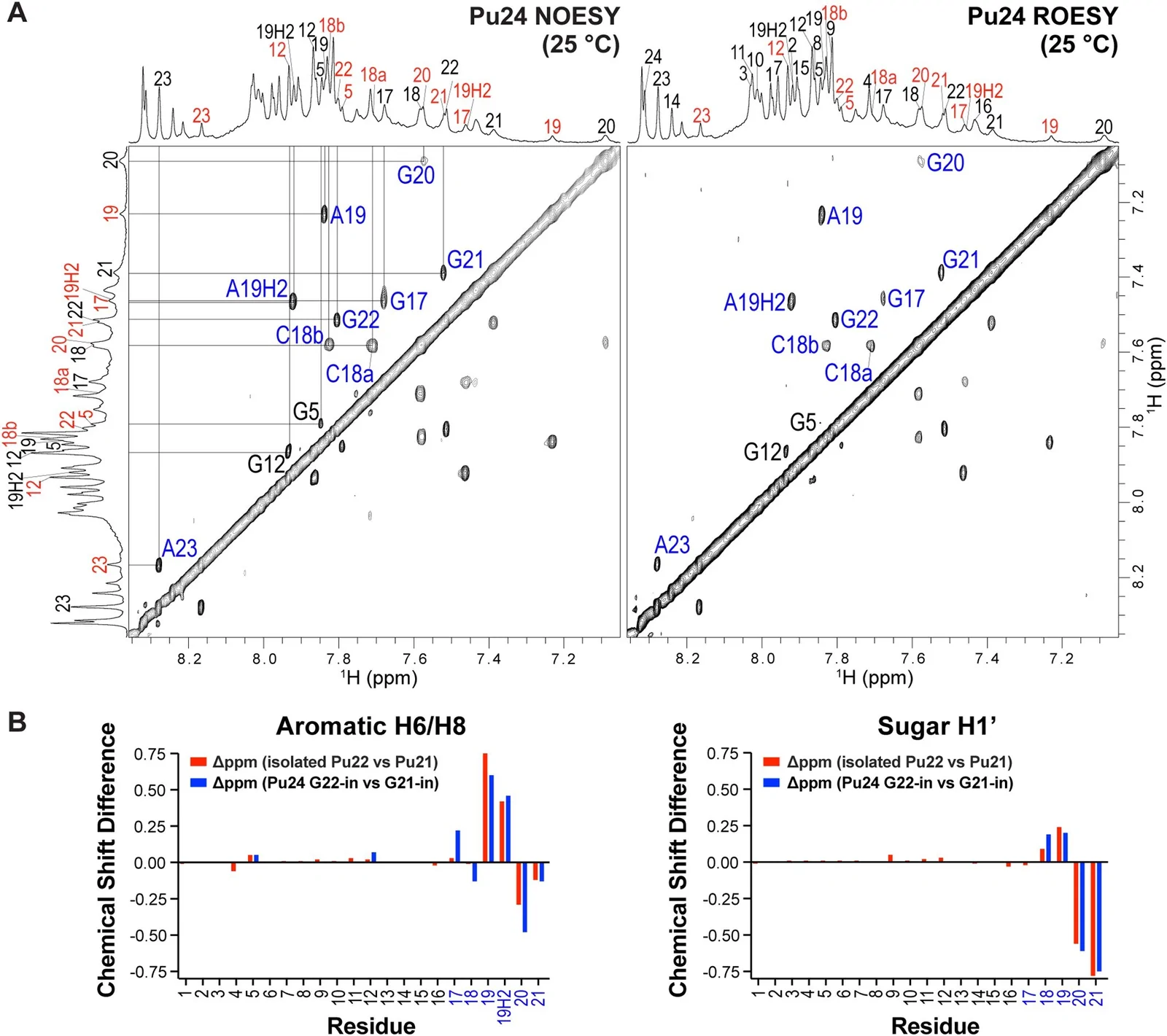
(**A**) Top: 1D ^1^H NMR spectrum aromatic region of the Pu24 sequence. Black = major G22-in species. Red = minor G21-in species. Bottom: 2D NOESY (left) and 2D ROESY (right) aromatic spectral region of Pu24. The mixing time for ROESY is 80 ms, while the mixing time for NOESY is 250 ms. Exchange cross peaks are observed in both NOESY and ROESY spectra, with each exchange cross peak showing connection between the major (black) and minor (red) species. Exchange cross peaks involving the 3′ snapback loop are labeled in blue. Note that C18H6 has two exchange peaks. NMR sample: 1 mM DNA, 100 mM K^+^, pH 7, 25°C, 10% D_2_O/90% H_2_O. (**B**) Plots of aromatic and sugar H1′ proton chemical shift differences (Δppm) between Pu22 and Pu21 (red) as well as between G22-in and G21-in species in Pu24 (Δppm of C18H6 is based on C18H6a).

**Figure 5.**
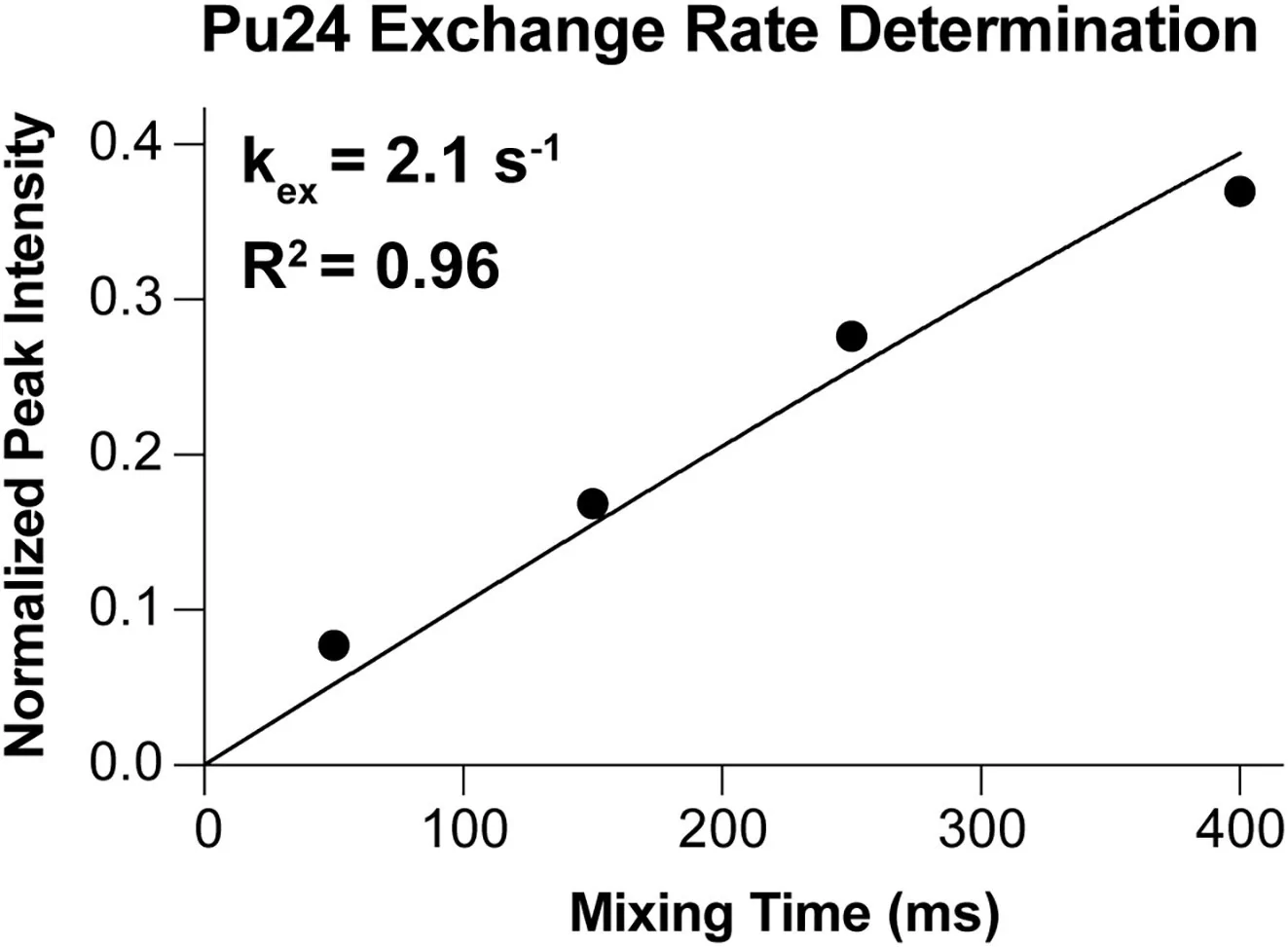
Exchange rate determination of the G22-in and G21-in species in Pu24, based on the isolated A23H8 exchange cross peak. The A23H8 NOESY exchange cross peak showed linear build up with increasing mixing times (50, 150, 250, 400 ms) at 25°C. NMR sample: 1 mM DNA, 100 mM K^+^, pH 7, 25°C, 10% D_2_O/90% H_2_O.

**Figure 6.**
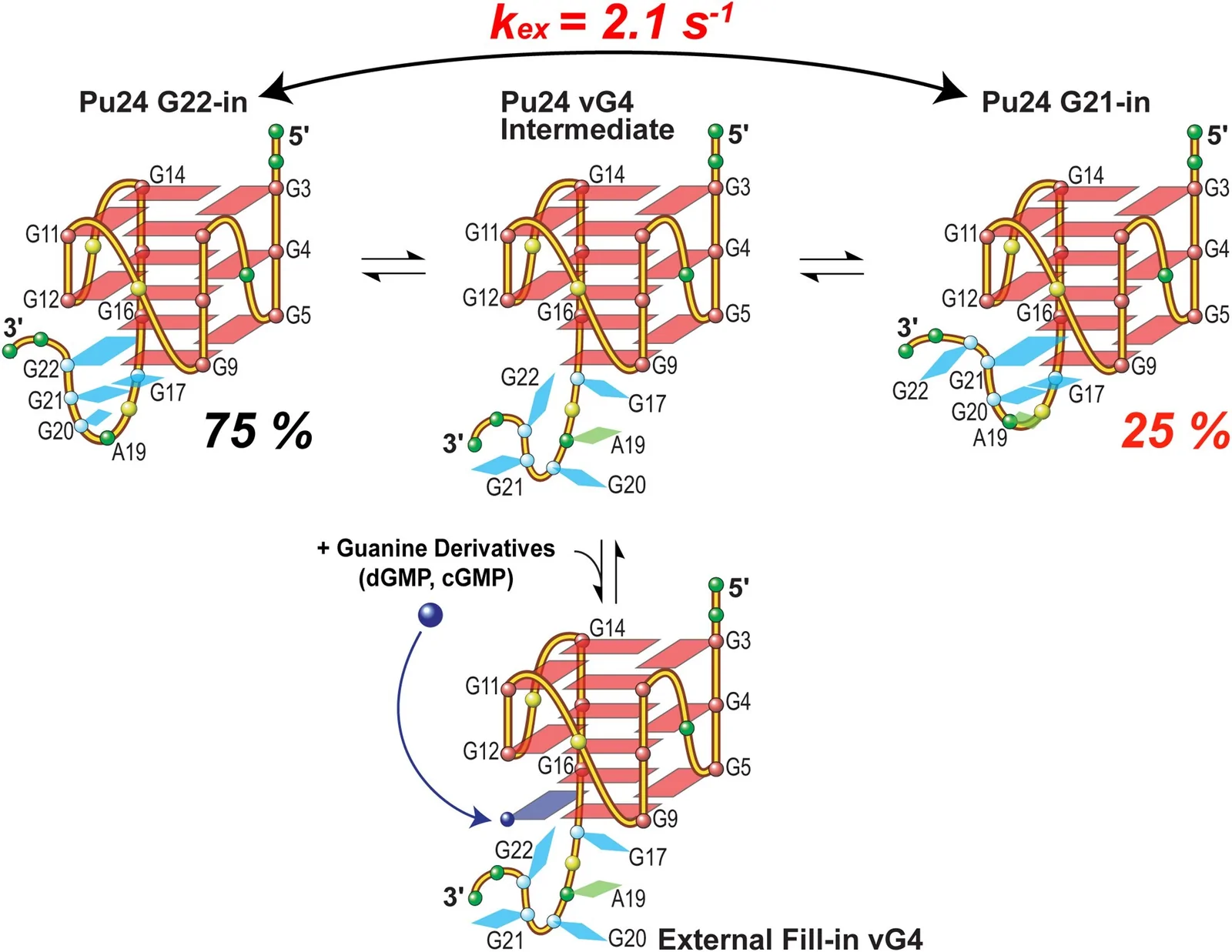
Folding equilibrium of the major PDGFR-β G4 in the extended Pu24 sequence. G22- and G21-filled-in intramolecular vG4s coexist in equilibrium and interconvert through a vG4 open state that can be filled-in by external guanine derivatives. The interconversion timescale is ∼500 ms.

Intriguingly, the NOESY spectra of Pu24 display additional cross peaks not attributable to NOE cross peaks of the G22-fill-in structure, as clearly shown in the diagonal regions corresponding to imino, aromatic, and sugar H1′ protons (Fig. 4A and Supplementary Fig. S17). Given that Pu24 contains two interconverting species, these new cross peaks likely represent exchange cross peaks between the G22-fill-in and G21-fill-in G4s. To confirm this, we performed a 2D ROESY (Rotating Frame Overhauser Effect Spectroscopy) experiment of Pu24. Whereas NOE and exchange cross peaks have the same phase in NOESY spectra, they exhibit opposite phases in ROESY experiments, allowing unambiguous identification of the exchange cross peaks (Fig. 4A and Supplementary Fig. S17) [46]. Based on the exchange cross peaks, the minor species of the G21-fill-in G4 can be assigned (Fig. 4B and Supplementary Fig. S18). To determine the exchange rate of the equilibrating G22-in and G21-in conformers in Pu24, we collected a series of NOESY spectra for Pu24 with increasing mixing times (50, 150, 250, 400 ms) at 25°C. Using the well-isolated A23H8 exchange peak (Fig. 4A), a linear buildup at increasing mixing times revealed an exchange rate (*k*_ex_) of 2.1 s^−1^ (Fig. 5). Therefore, in the extended Pu24 sequence, G22-in and G21-in G4s coexist and interconvert with a timescale of ∼500 ms (Fig. 6). The exchange cross peaks involve residues from G17 to G22, encompassing the 3′ snap-back loop, the fill-in guanine, and the 3′-flanking residues. This suggests the interconversion of the 3′ snap-back loop, while the vG4 core remains intact.

Using the isolated A23H8 peaks from the two species in Pu24 at 25°C (Supplementary Fig. S18), we also quantified the populations by peak integration. The G22-fill-in G4 accounts for 75% (major), while the G21-fill-in structure represents 25% (minor) (Fig. 6). This is consistent with the predominance of Pu22-like cross peaks in the Pu24 NOESY spectrum (Supplementary Figs S14 and S15), indicating that the presence of additional 3′-flanking residues favors the G22-fill-in structure (Fig. 6), albeit the similar thermal stability of the truncated Pu22 and Pu21 (Fig. 1B). We have previously conducted DMS footprinting of the extended PDGFR-β promoter sequence Pu38, which contains the native 3′ flanking extension (AGGGTGGA) (Supplementary Fig. S19) [15]. The footprinting result shows that G20 is fully cleaved indicating its location in the loop. In contrast, G22 is clearly protected from DMS access indicating its involvement in the G-tetrad core, while G21 shows partial protection. This is consistent with the structures we determined with G22-fill-in being the major species (Fig. 6).

### Extended PDGFR-β promoter sequences can form vG4 structures and be filled-in by external dGMP

**Figure 7.**
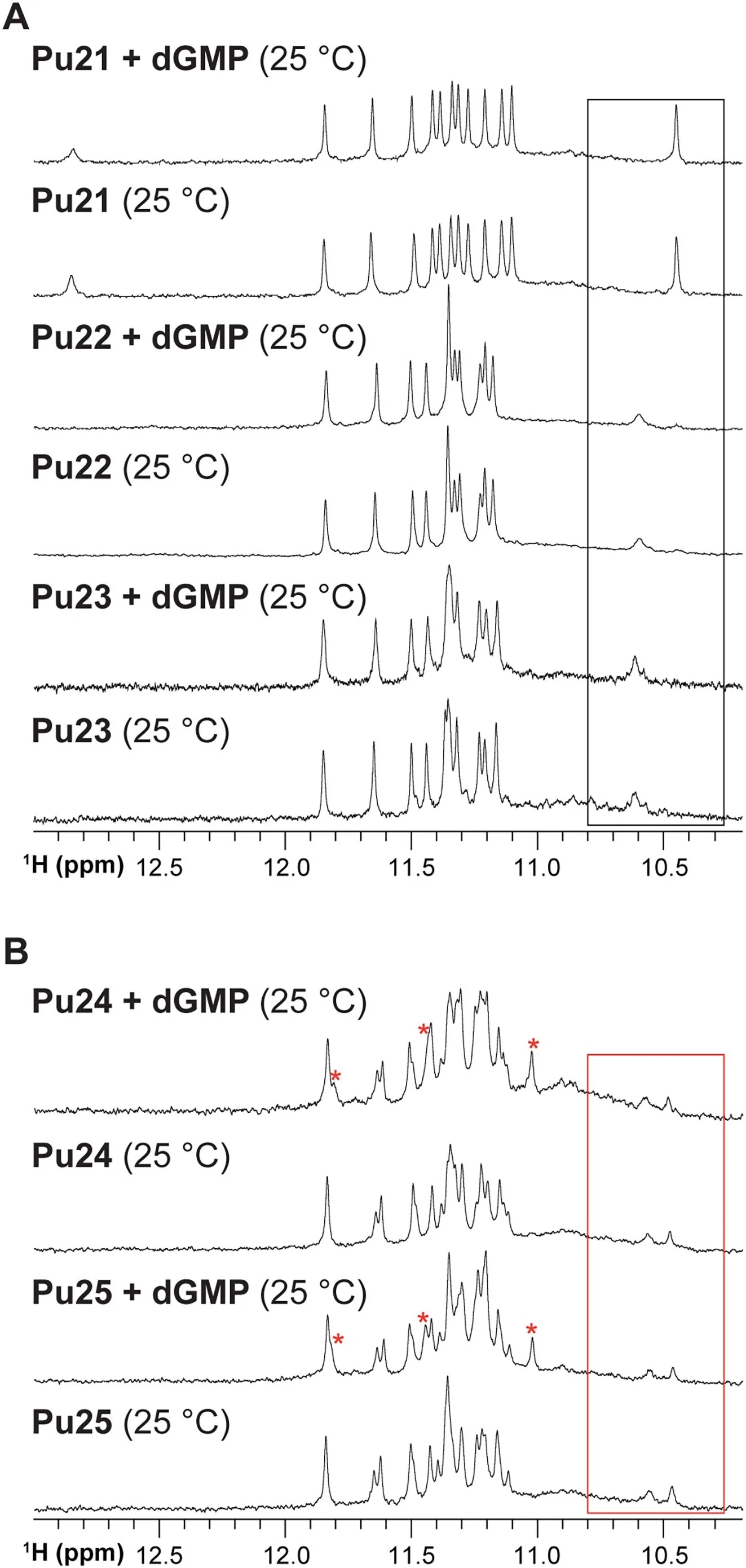
1D ^1^H NMR spectral imino regions of the (**A**) Pu21, Pu22, and Pu23 sequences as well as (**B**) Pu24 and Pu25 sequences, in the presence and absence of 40 equivalence dGMP. Pu21, Pu22, and Pu23 cannot be filled-in by dGMP, whereas Pu24 and Pu25 can be filled-in by dGMP as shown by new peaks (red asterisks) upon dGMP addition. Black box: Pu21, Pu22, and Pu23 adopt one major conformation and cannot be filled-in by dGMP. Red box: Pu24 and Pu25 adopt two equilibrating species, whose interconversion forms vG4 and enables external fill-in by dGMP. Sample condition: 150 μM DNA, 90 mM K^+^, pH 7, 25°C, 10% D_2_O/90% H_2_O.

The truncated Pu19 sequence, which lacks G-run V, forms a vG4 structure that can be filled in by external guanine derivatives such as dGMP (Supplementary Fig. S1) [27, 47]. The broken-strand G4 formed by Pu22 or Pu21 can be viewed as vG4 with a vacancy in the 3′-tetrad, which is intramolecularly filled by the 3′-terminal G22 or G21 from G-run V (Fig. 1C). However, addition of dGMP to the Pu22 sequence does not induce appreciable spectral changes, indicating that external dGMP cannot compete with the intramolecular fill-in by G22 (Fig. 7A, Pu22). A similar result is observed for the truncated Pu21 sequence, which also resists dGMP fill-in (Fig. 7A, Pu21). These findings suggest that the presence of a 3′-terminal G22 or G21 effectively prevents vG4 formation and external fill-in, likely due to the energetically favorable intramolecular fill-in facilitated by proximal and terminal positioning.

Interestingly, the melting temperature of Pu24 is significantly lower (*T*_m_ = 69°C) than that of the truncated Pu22 and Pu21 sequences (*T*_m_ = 80°C) (Fig. 1B). The reduced overall structural stability in the extended promoter sequence connects to the dynamic interconversion between the two fill-in G4s, which requires an open state vG4 that can be filled-in by external guanine derivatives. To test this hypothesis, we examined dGMP fill-in for Pu24. Indeed, addition of dGMP to Pu24 results in clear spectral changes (Fig. 7B, Pu24), indicating vG4 formation and external fill-in. We further tested Pu25, which contains three 3′-flanking residues, and found that it also readily accommodates dGMP fill-in (Fig. 7B, Pu25). In contrast, Pu23, with only one 3′-flanking residue, does not show structural interconversion or dGMP fill-in (Fig. 7A, Pu23).

Taken together, our results suggest that a minimum 3′-extension of two residues is required for the formation of two interconverting fill-in structures (Fig. 6). This requirement likely arises because the intramolecular fill-in guanine becomes more dynamic with extended flanking sequences and transiently detaches, leading to the formation of a vG4 open state. This transient vG4 structure can then be filled-in by external guanine derivatives, as demonstrated by Zheng *et al*. in cancer cells [29]. While interconversion between the two broken-strand G4s is an intrinsic property of the PDGFR-β promoter, guanine metabolite binding (affinity in the micromolar range [27]) is expected to stabilize (“lock”) the vG4 conformation, shifting the equilibrium toward this state. Significantly, intracellular GTP concentrations (∼0.1–2 mM) are frequently elevated in rapidly proliferating cancer cells [48–51].

## Conclusion

The PDGFR-β promoter vG4 represents a promising target for selective drug intervention and transcriptional regulation. In this study, we elucidated the structural mechanism by which vG4 forms within the biologically relevant, extended PDGFR-β promoter sequence. We discovered that the intramolecular fill-in guanines of the broken-strand G4 become dynamic in an extended promoter context, transiently detaching to form a vG4 intermediate. This open-state can then be filled-in either by alternative intramolecular guanines or by external guanine derivatives such as dGMP (Fig. 6).

We determined high-resolution NMR structures of the two intramolecular fill-in G4s formed by the extended PDGFR-β promoter sequence. In K^+^ solution, both structures share the same vG4 core but differ in the fill-in guanine. These fill-ins are stabilized by a unique reverse-Hoogsteen G:G capping base pair. The snap-back structures still form with non-terminal Gs suggesting they can exist in the genomic context with longer flanking segments. Further NMR analyses revealed that these two conformers interconvert through a vG4 on the millisecond timescale, exchanging the fill-in guanine without global refolding of the vG4 core. Our findings thus reveal a unique vG4-forming mechanism intrinsic to the sequence and dynamic behavior of the PDGFR-β promoter. This study establishes the structural basis for selectively targeting PDGFR-β vG4s, offering a promising avenue for therapeutic intervention in diseases associated with aberrant PDGFR-β expression.

## Supplementary Material

gkag843_Supplemental_File

## Data Availability

Coordinates of the major PDGFR-β Pu22 and Pu21 G4 structures were deposited in the Protein Data Bank (9P9X, 9P9Y). Chemical shift data were deposited in the Biological Magnetic Resonance Data Bank (31252, 31253).
