# Supplementary material for: Structures and dynamics of the major G-quadruplex in the human PDGFR-β gene promoter: insights into vacancy G-quadruplex formation
Source: Nucleic Acids Res. 2026 Sep 4;54(16):gkag843. doi: 10.1093/nar/gkag843 (PMC13542729; doi:10.1093/nar/gkag843)
Supplement: Supplementary file 1 [file gkag843_supplemental_file.pdf]

Supplementary Information

Structures and Dynamics of the Major G-Quadruplex in the Human PDGFR-β Gene Promoter: Insights into Vacancy G-Quadruplex Formation

Yichen Han[1], Jonathan Dickerhoff[1], and Danzhou Yang[1,2*]

[1] Borch Department of Medicinal Chemistry and Molecular Pharmacology, College of Pharmacy, Purdue University, West Lafayette, IN 47906, USA
[2] Purdue Institute for Cancer Research, James Tarpo Jr. and Margaret Tarpo Department of Chemistry, Purdue Institute for Drug Discovery, Purdue University, West Lafayette, IN 47906, USA
* To whom correspondence should be addressed. Email: yangdz@purdue.edu

Table of Contents

Figures	Contents	Pages
Figure S1	1D NMR titration of Pu19 with dGMP	3
Figure S2	1D NMR imino region of Pu25, Pu24, Pu23, Pu22, Pu21, and Pu20	4 – 5
Figure S3	2D NOESY H8-H8 spectral regions of Pu22	6 – 8
Figure S4	2D NOESY H8-H8 spectral regions of Pu21	9 – 10
Figure S5	2D NOESY H8-H1′ spectral regions of Pu22	11 – 13
Figure S6	2D NOESY H8-H1′ spectral region of Pu21	14 – 15
Figure S7	CD data for Pu25, Pu24, Pu23, Pu22, Pu21, Pu20, and Pu19 sequences	16
Figure S8	Schematic NOE summary for Pu22 G-quadruplex	17
Figure S9	Schematic NOE summary for Pu21 G-quadruplex	18
Figure S10	2D ^{1}H-^{13}C HSQC of Pu22 and Pu21	19 – 20
Figure S11	Ensemble of lowest-energy structures for Pu22 G-quadruplex	21
Figure S12	Ensemble of lowest-energy structures for Pu21 G-quadruplex	22
Figure S13	1D NMR and CD data of Pu22 and P21 mutants	23
Figure S14	2D NOESY H1-H9 spectral region of Pu24	24
Figure S15	2D NOESY H8-H1′ spectral regions of Pu24	25
Figure S16	2D ^{1}H-^{13}C HSQC of Pu24	26
Figure S17	2D NOESY and/or ROESY H1′- H1′, H1-H1, and H8-H8 spectral regions of Pu24	27 – 28
Figure S18	1D NMR imino and aromatic regions of Pu24, Pu22, and Pu21	29
Figure S19	DMS footprinting data of full-length PDGFR-β promoter NHE Pu38	30

Tables	Contents	Pages
Table S1	Oligonucleotide sequences and melting temperatures	31
Table S2-5	Chemical shifts of Pu22 and Pu21	32 – 35
Table S6-9	Restraint tables of Pu22 and Pu21	36 – 39
Table S10	Structural statistics of Pu22 and Pu21 G-quadruplex NMR structures	40

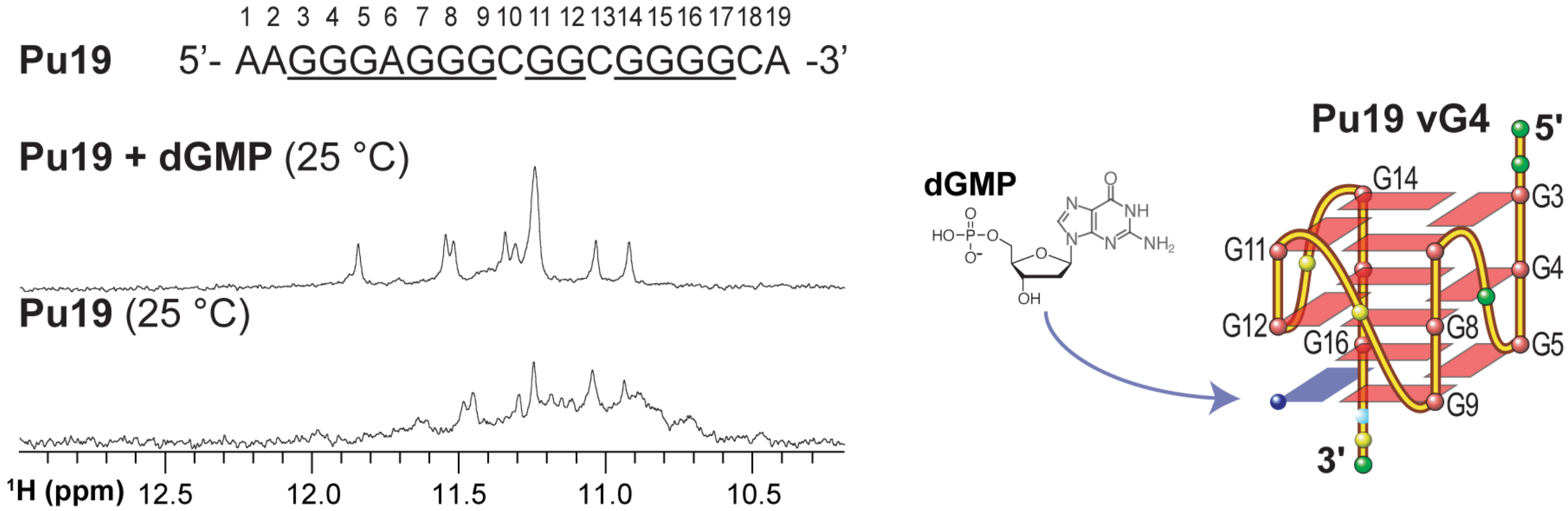

Figure S1. Top: Sequence of Pu19 vG4. Left: 1D ^{1}H NMR spectra showing imino regions of Pu19 in presence and absence of 40 equivalence dGMP. Sample condition: 150 μM DNA, 90 mM K^{+}, pH 7, 25 °C, 10 % D_2O/90 % H_2O. Right: Schematic folding of the Pu19 vG4 with dGMP fill-in.

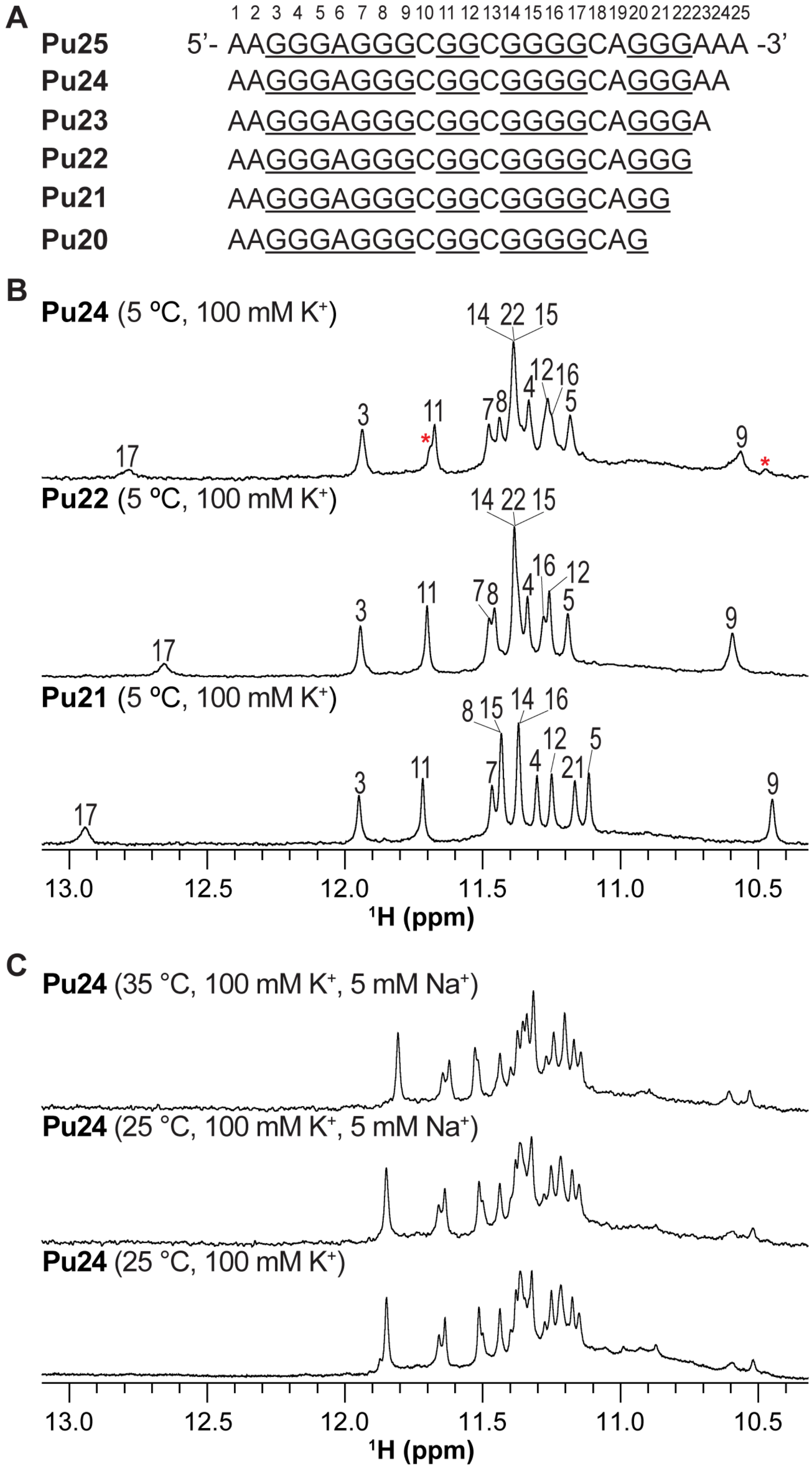

A
1 2 3 4 5 6 7 8 9 10 11 12 13 14 15 16 17 18 19 20 21 22 23 24 25
Pu25 5'- AAGGGAGGGCGGCGGGGCAGGGAAA -3'
Pu24 AAGGGAGGGCGGCGGGGCAGGGAA
Pu23 AAGGGAGGGCGGCGGGGCAGGGA
Pu22 AAGGGAGGGCGGCGGGGCAGGG
Pu21 AAGGGAGGGCGGCGGGGCAGG
Pu20 AAGGGAGGGCGGCGGGGCAG
B
Pu24 (5 ºC, 100 mM K+)
Pu22 (5 ºC, 100 mM K+)
Pu21 (5 ºC, 100 mM K+)
13.0
12.5
12.0
11.5
11.0
10.5
1H (ppm)
C
Pu24 (35 °C, 100 mM K+, 5 mM Na+)
Pu24 (25 °C, 100 mM K+, 5 mM Na+)
Pu24 (25 °C, 100 mM K+)
13.0
12.5
12.0
11.5
11.0
10.5
1H (ppm)

D

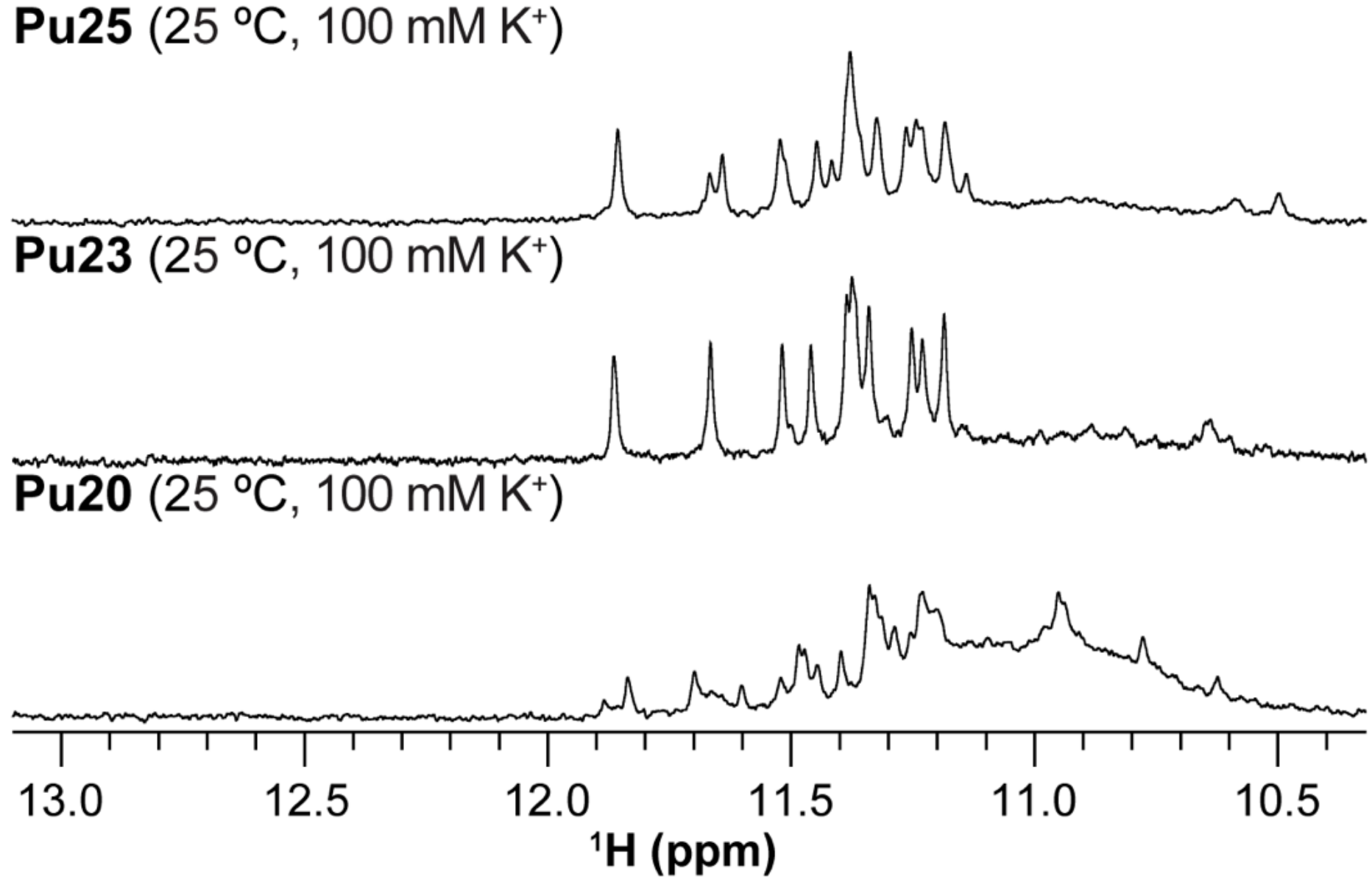

Figure S2. (A) Sequences of Pu25, Pu24, Pu23, Pu22, Pu21, and Pu20. B) 1D ^{1}H NMR spectra showing the imino region of Pu24, Pu22, and Pu21 at 5 °C. Asterisks: Minor species of Pu24. C) 1D ^{1}H NMR spectra showing the imino region of Pu24 at 25 °C and 35 °C as well as in the presence of additional 5 mM Na^+. D) 1D ^{1}H NMR spectra showing imino region of Pu25, Pu23, and Pu20 at 25 °C. Sample condition (unless specified for Na^+): 150 µM DNA, 100 mM K^+, pH 7, 10 % D_2O/90 % H_2O.

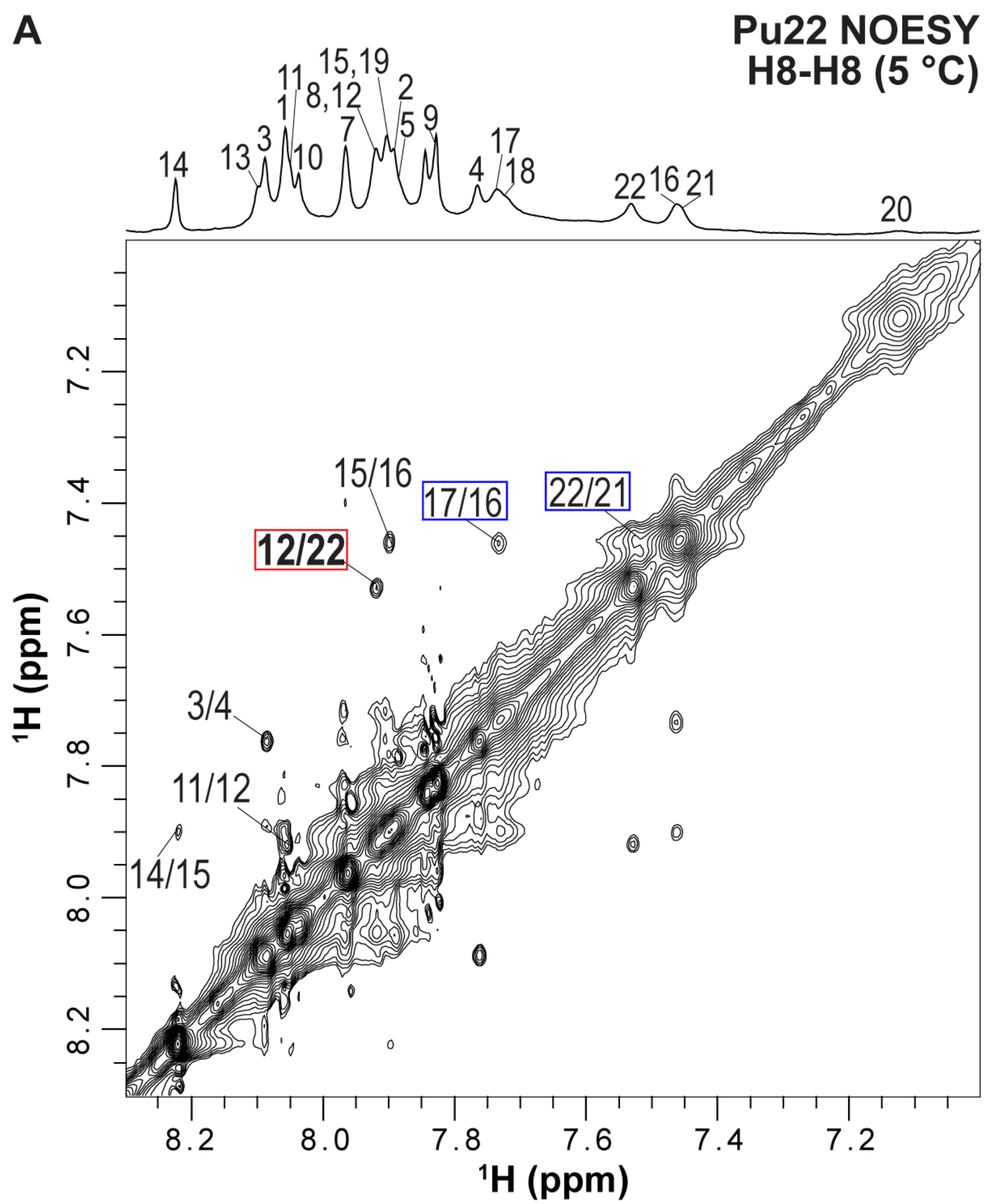
A
Pu22 NOESY
H8-H8 (5 °C)
14
13
3
1
11
10
8,12
15,19
7
2
5
9
4
17
18
22
16
21
20
15/16
17/16
22/21
12/22
3/4
11/12
14/15
7.2
7.4
7.6
7.8
8.0
8.2
¹H (ppm)
¹H (ppm)

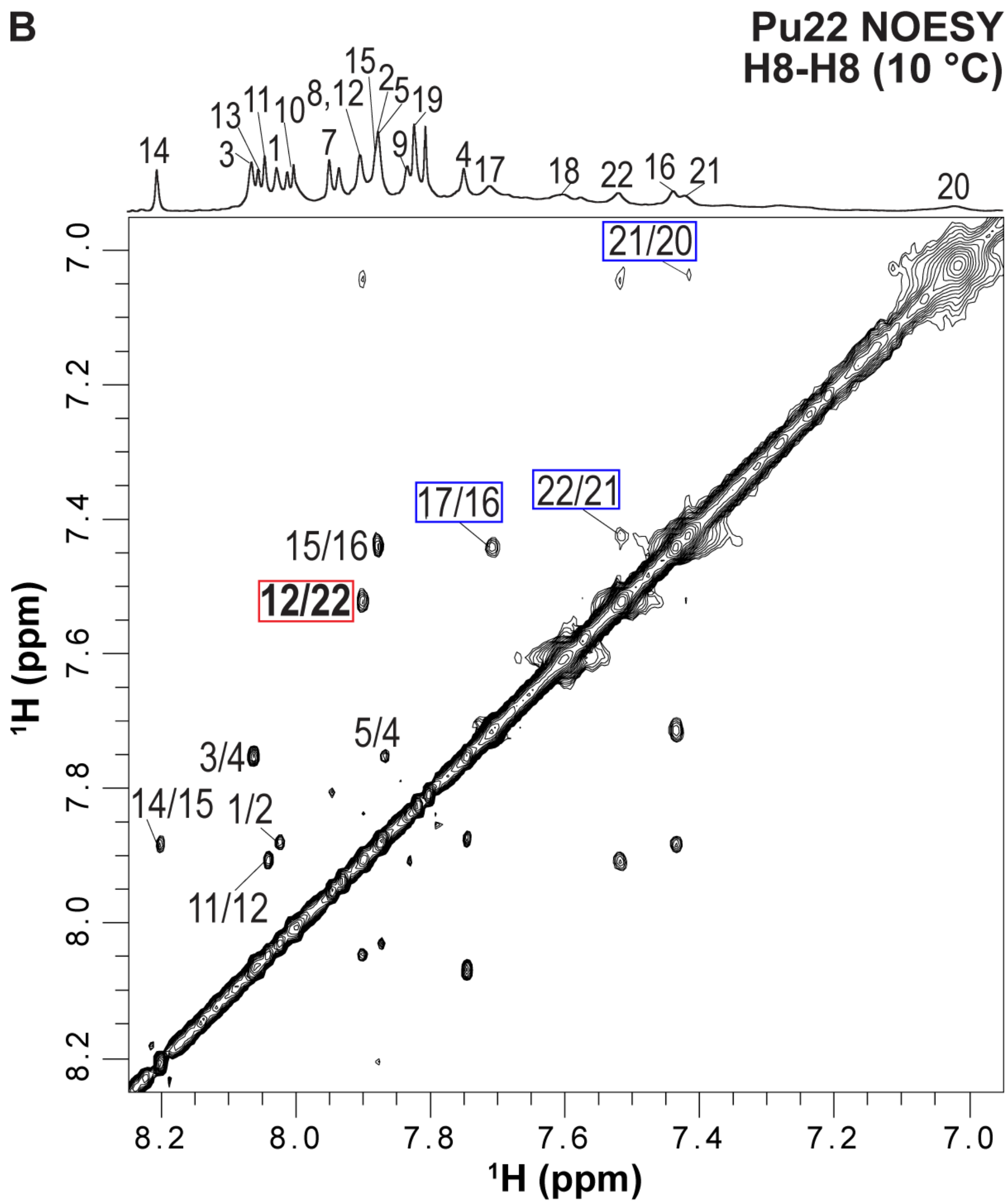

B
Pu22 NOESY
H8-H8 (10 °C)
14
13
3
11
1
10
8,12
15
2
5
19
7
9
4
17
18
22
16
21
20
21/20
17/16
22/21
15/16
12/22
5/4
3/4
14/15
1/2
11/12
¹H (ppm)
¹H (ppm)

C

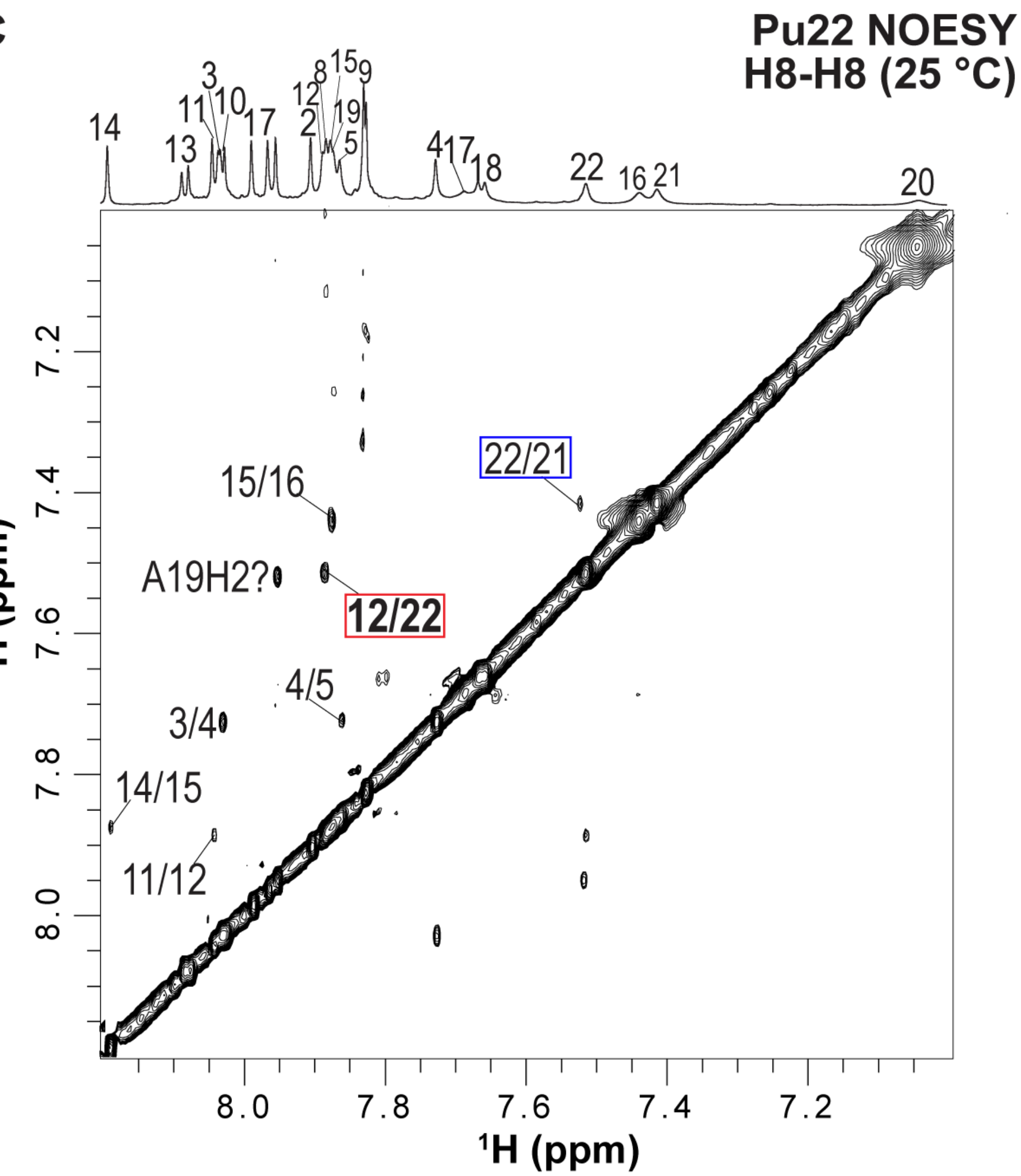

Figure S3. 2D NOESY H8-H8 spectral regions of the major PDGFR-β Pu22 G-quadruplex at A) 5 °C, B) 10 °C, and C) 25 °C. Red box = G22H8-G12H8 NOE defining G22 fill-in. Blue box = 3′-capping NOEs showing G17:G21 base pair stacking on the 3′-tetrad. NOESY were measured with a mixing time of 250 ms. Sample condition: 1 mM DNA, 100 mM K^+, pH 7, 10 % D_2O/90 % H_2O.

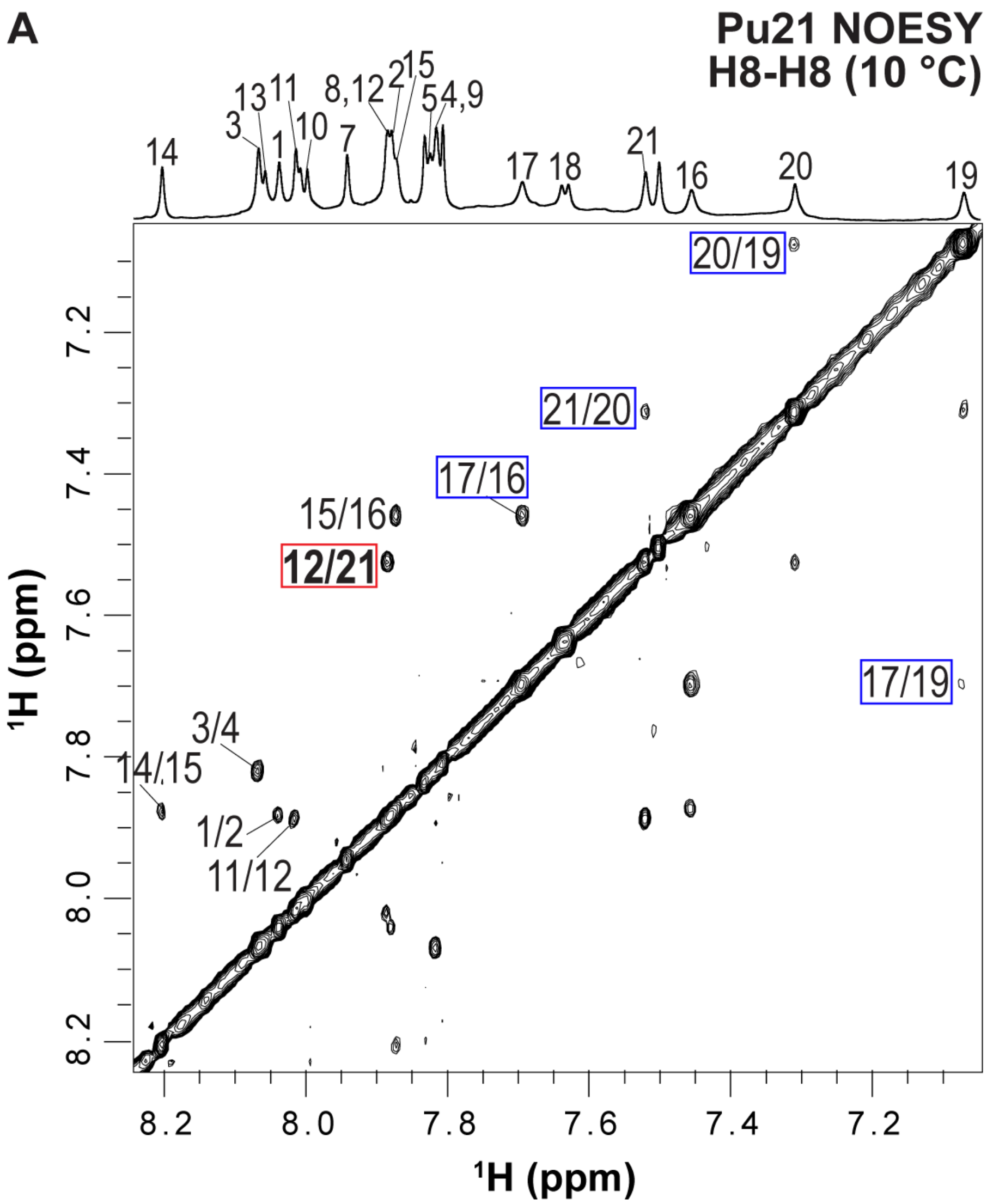

A
Pu21 NOESY
H8-H8 (10 °C)
14
3
13
1
11
10
7
8,12
2
15
5
4,9
17
18
21
16
20
19
20/19
21/20
17/16
15/16
12/21
17/19
3/4
14/15
1/2
11/12
7.2
7.4
7.6
7.8
8.0
8.2
¹H (ppm)
8.2
8.0
7.8
7.6
7.4
7.2
¹H (ppm)

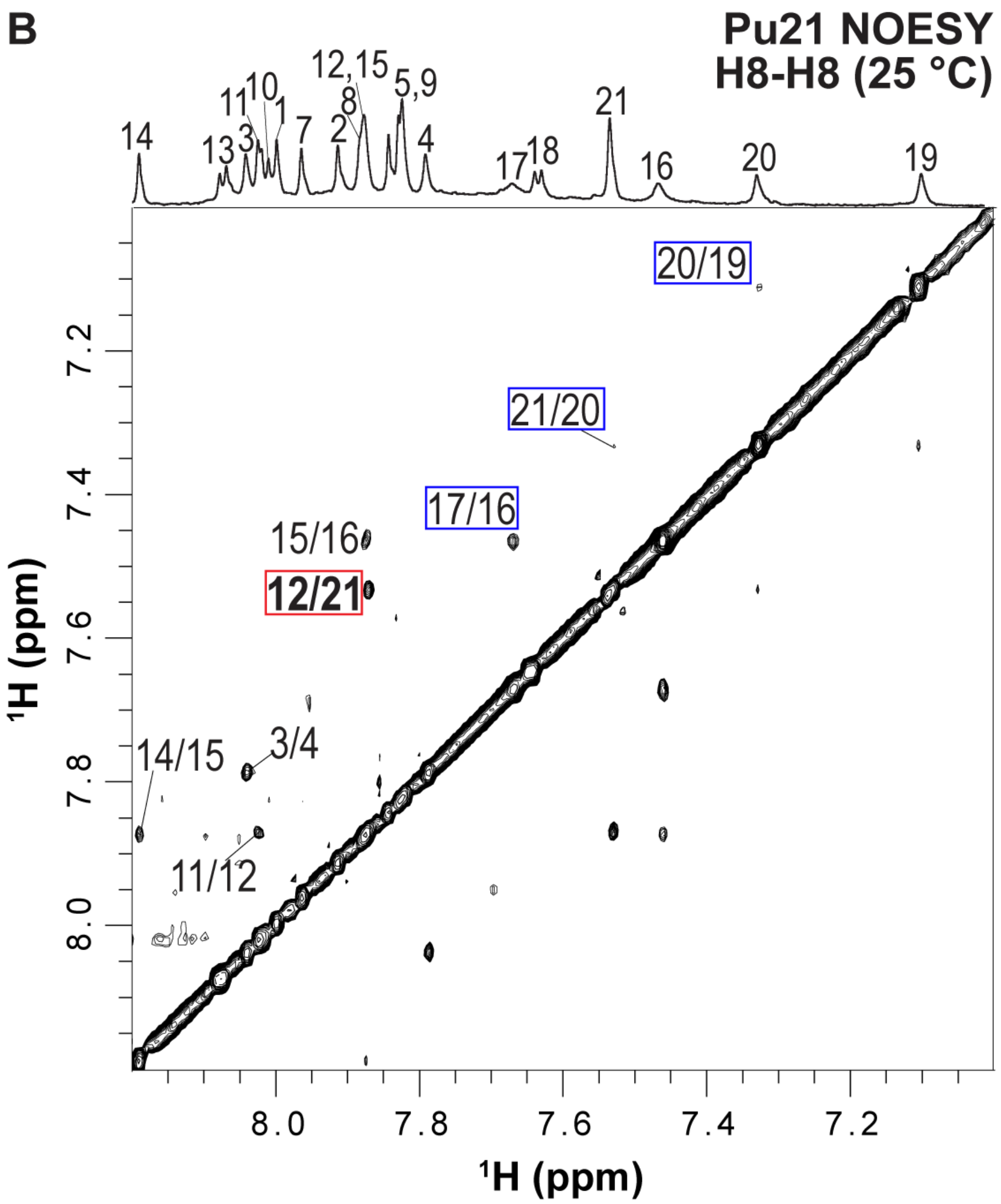

Figure S4. 2D NOESY H8-H8 spectral regions of the major PDGFR-β Pu21 G-quadruplex at A) 10 °C and B) 25 °C. Red box = G21H8-G12H8 NOE defining G21 fill-in. Blue box = 3′-capping NOEs showing G17:G20 base pair stacking on the 3′-tetrad. NOESY were measured with a mixing time of 250 ms. Sample condition: 1 mM DNA, 100 mM K^+, pH 7, 10 % D_2O/90 % H_2O.

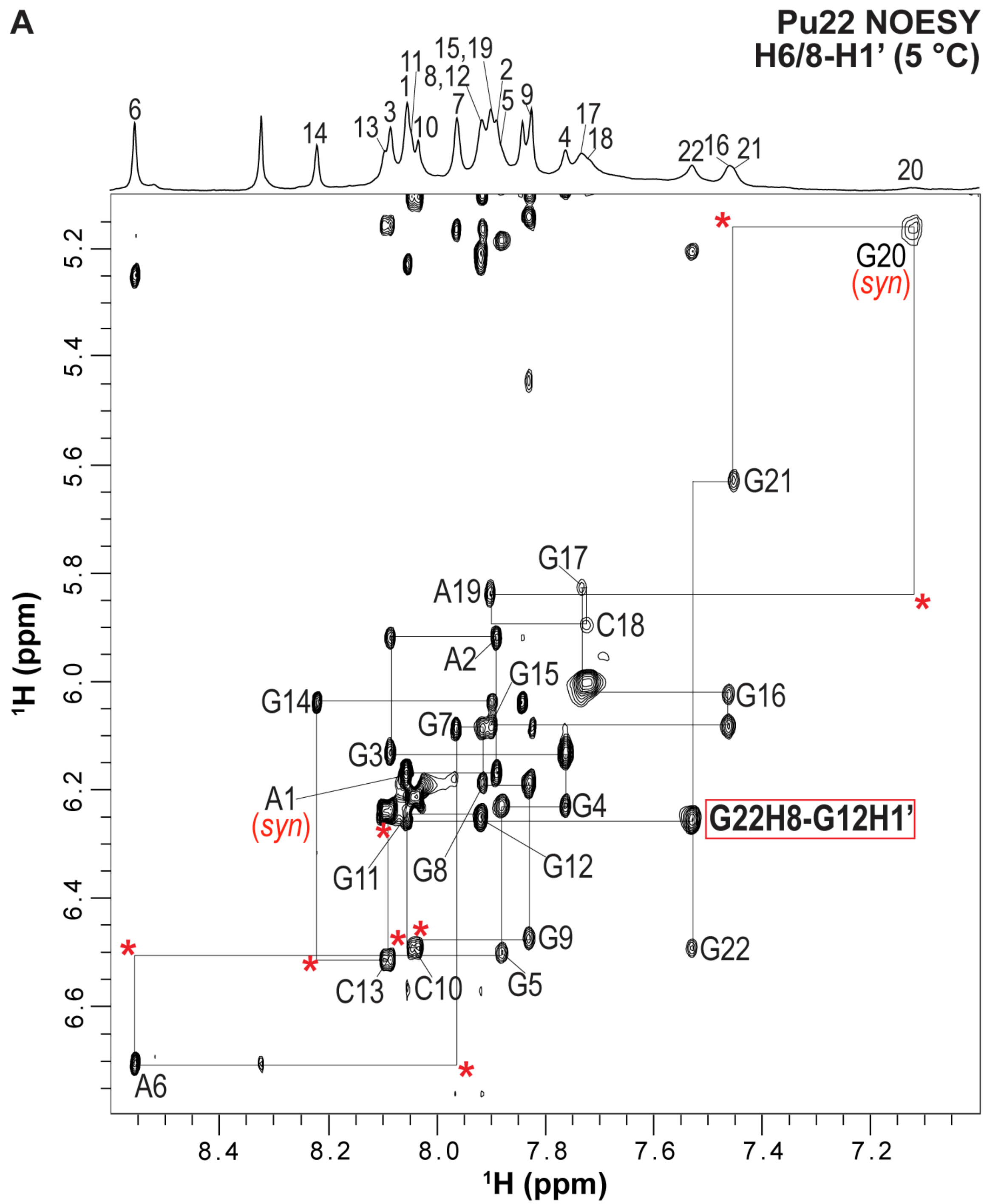
A
Pu22 NOESY
H6/8-H1' (5 °C)
G20
(syn)
G21
G17
A19
C18
A2
G15
G14
G16
G7
G3
A1
(syn)
G4
G22H8-G12H1'
G11
G8
G12
G9
G22
C13
C10
G5
A6
¹H (ppm)
¹H (ppm)

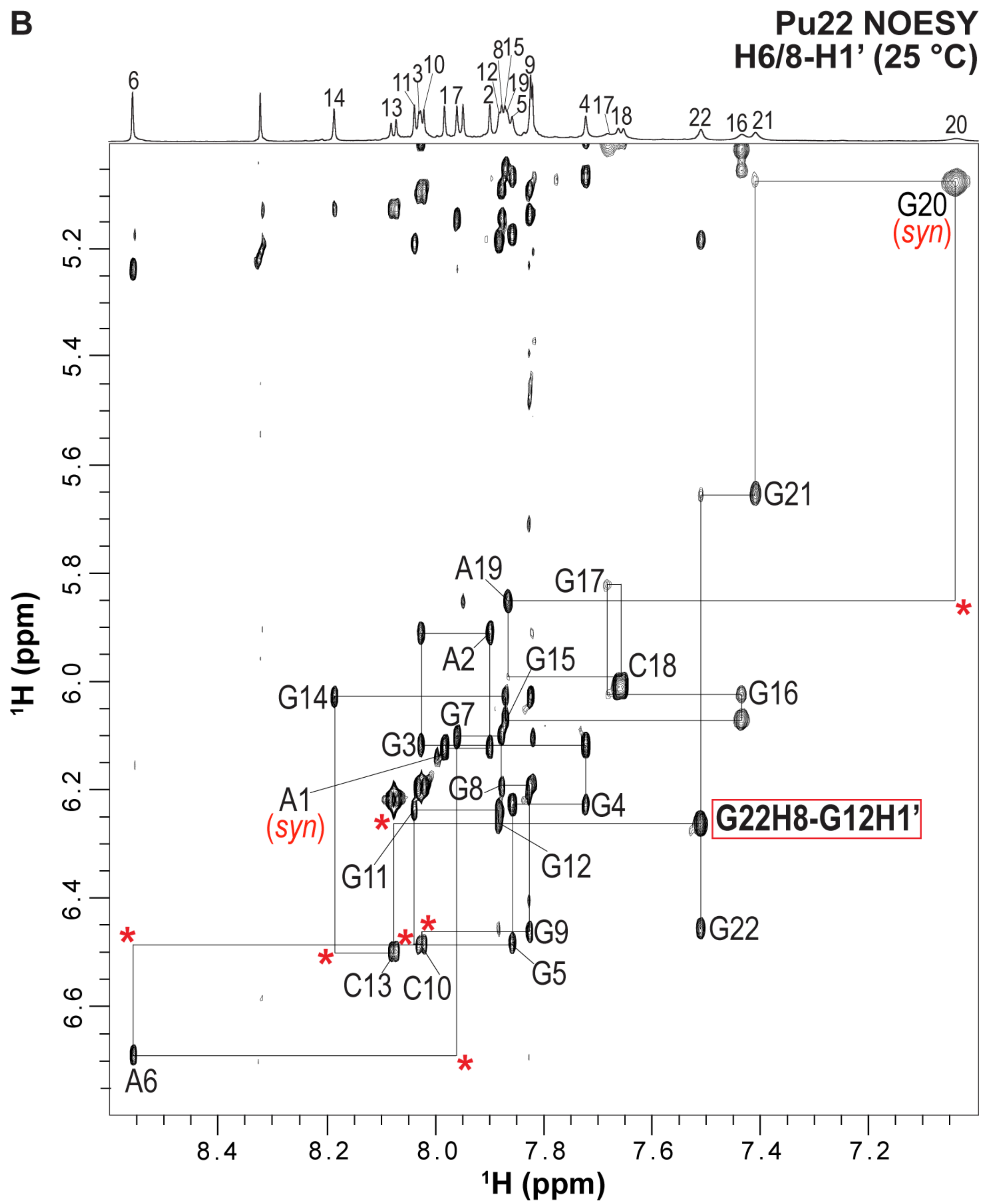

B
Pu22 NOESY
H6/8-H1' (25 °C)
6
14
13
11
3
10
17
12
2
8
15
19
9
5
4
17
18
22
16
21
20
G20
(syn)
G21
A19
G17
A2
G15
C18
G14
G16
G7
G3
A1
(syn)
G8
G4
G22H8-G12H1'
G11
G12
G9
G22
C13
C10
G5
A6
¹H (ppm)
¹H (ppm)
5.2
5.4
5.6
5.8
6.0
6.2
6.4
6.6
8.4
8.2
8.0
7.8
7.6
7.4
7.2

C

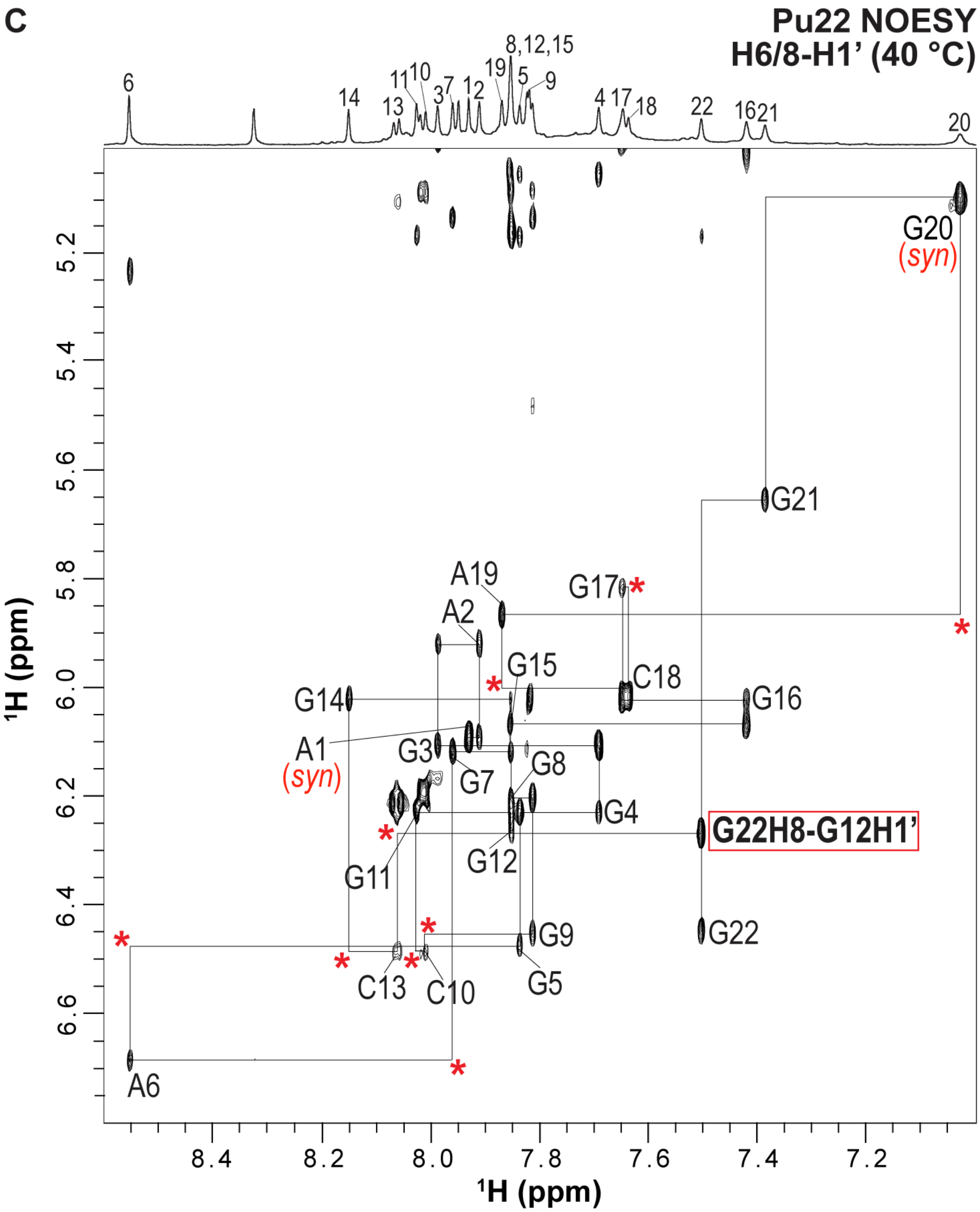

Figure S5. 2D NOESY H6/8-H1′ spectral regions of the major PDGFR-β Pu22 G-quadruplex at A) 5 °C, B) 25 °C, and C) 40 °C. Sequential NOE contacts are traced by solid lines. Red box = G22H8-G12H1′ NOE defining G22 fill-in. Red label = strong G20H8-H1′ NOE suggesting G20 *syn* conformation. Missing NOE cross peaks are marked with an asterisk. NOESY were measured a mixing time of 250 ms. Sample condition: 1 mM DNA, 100 mM K^+, pH 7, 10 % D_2O/90 % H_2O.

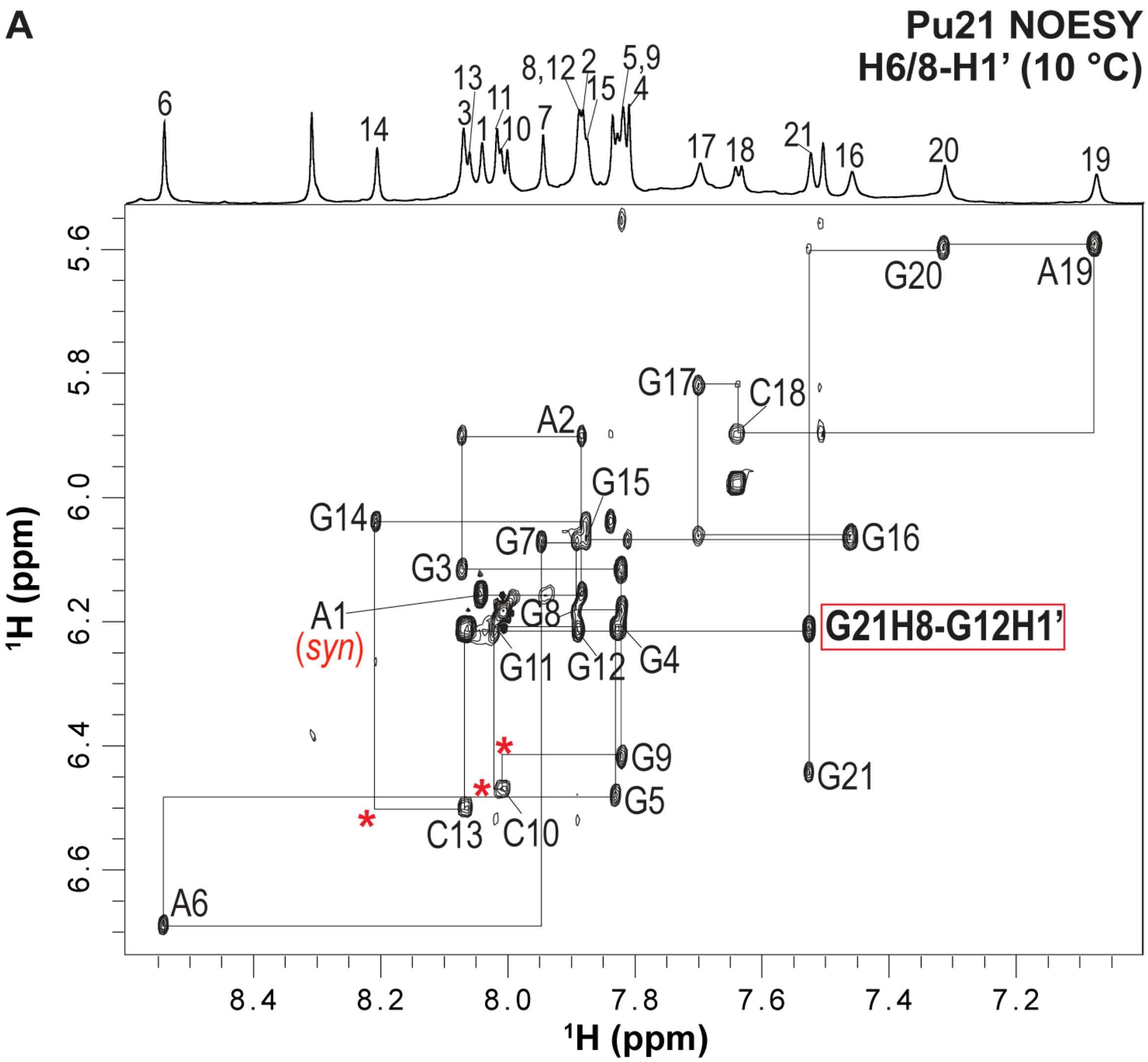
A
Pu21 NOESY
H6/8-H1' (10 °C)
6
14
13
3
1
11
10
7
8,12
2
15
5,9
4
17
18
21
16
20
19
A2
G17
C18
A19
G20
G15
G14
G16
G7
G3
A1
(syn)
G8
G11
G12
G4
G21H8-G12H1'
G9
G21
G5
C13
C10
A6
¹H (ppm)
5.6
5.8
6.0
6.2
6.4
6.6
8.4
8.2
8.0
7.8
7.6
7.4
7.2
¹H (ppm)

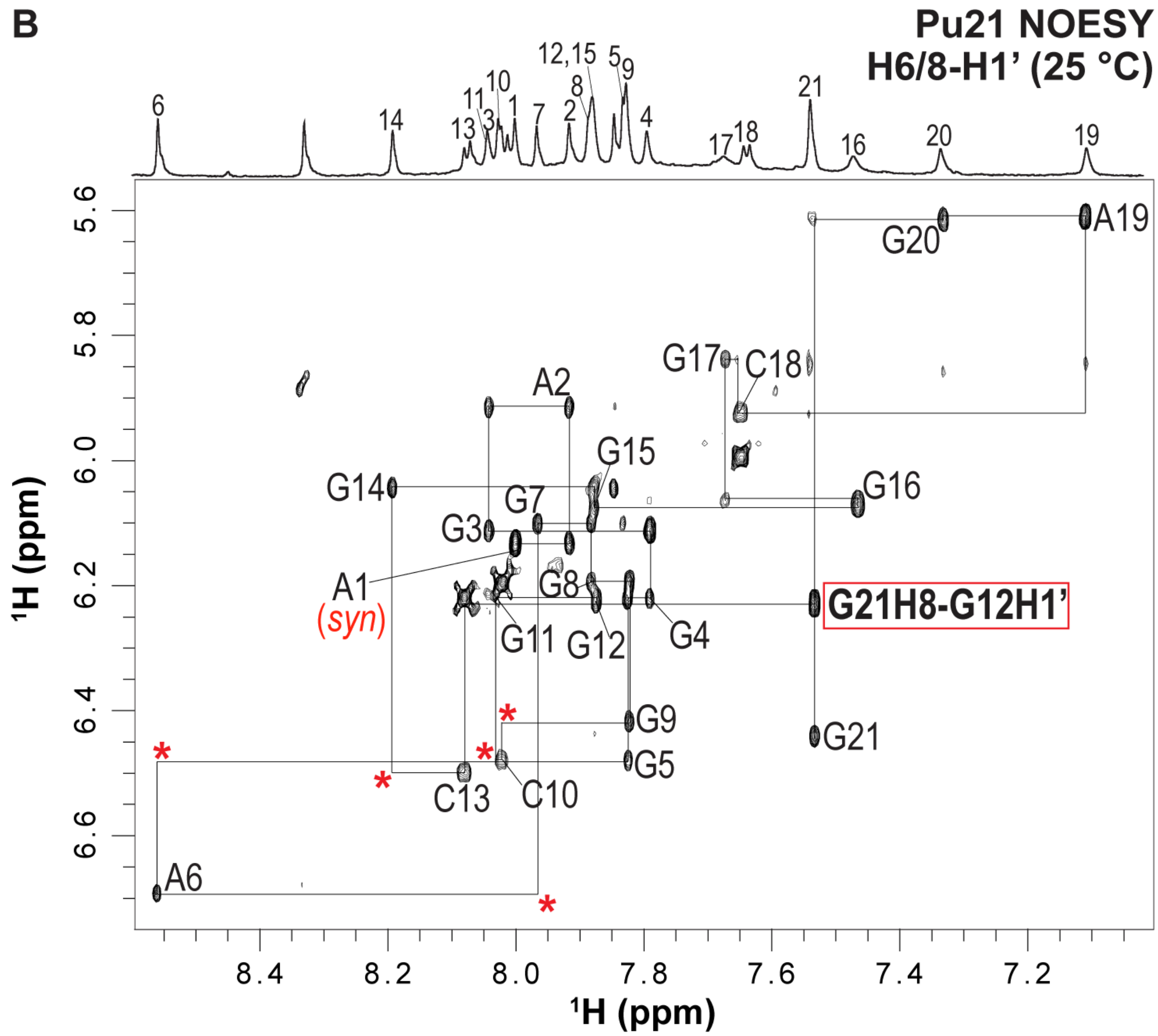

Figure S6. 2D NOESY H6/8-H1′ spectral regions of the major PDGFR-β Pu21 G-quadruplex at A) 10 °C and B) 25 °C. Sequential NOE contacts are traced by solid lines. Red box = G21H8-G12H1′ NOE defining G21 fill-in. Missing NOE cross peaks are marked with an asterisk. NOESY were measured a mixing time of 250 ms. Sample condition: 1 mM DNA, 100 mM K^+, pH 7, 10 % D_2O/90 % H_2O.

		T_m (ºC)
	1 2 3 4 5 6 7 8 9 10 11 12 13 14 15 16 17 18 19 20 21 22 23 24 25	
**Pu25**	5'- AAGGGAGGGCGGCGGGGCAGGGAAA -3'	69
**Pu24**	AAGGGAGGGCGGCGGGGCAGGGAA	69
**Pu23**	AAGGGAGGGCGGCGGGGCAGGGA	75
**Pu22**	AAGGGAGGGCGGCGGGGCAGGG	80
**Pu21**	AAGGGAGGGCGGCGGGGCAGG	80
**Pu20**	AAGGGAGGGCGGCGGGGCAG	67
**Pu19**	AAGGGAGGGCGGCGGGGCA	66

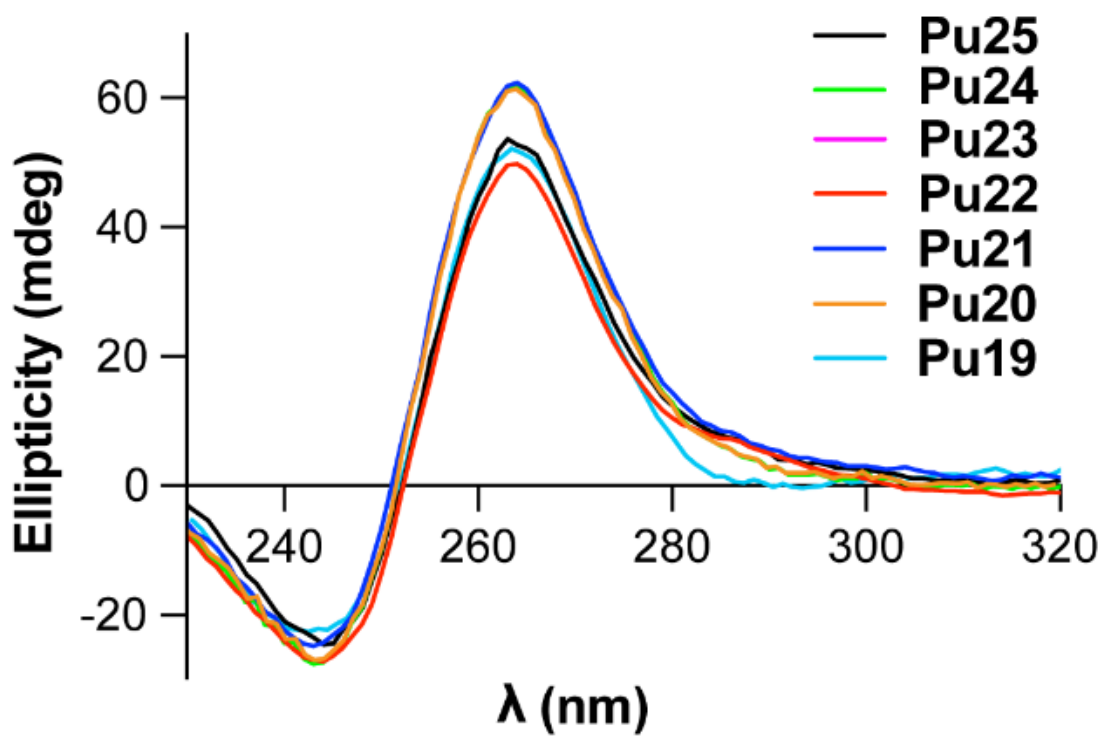

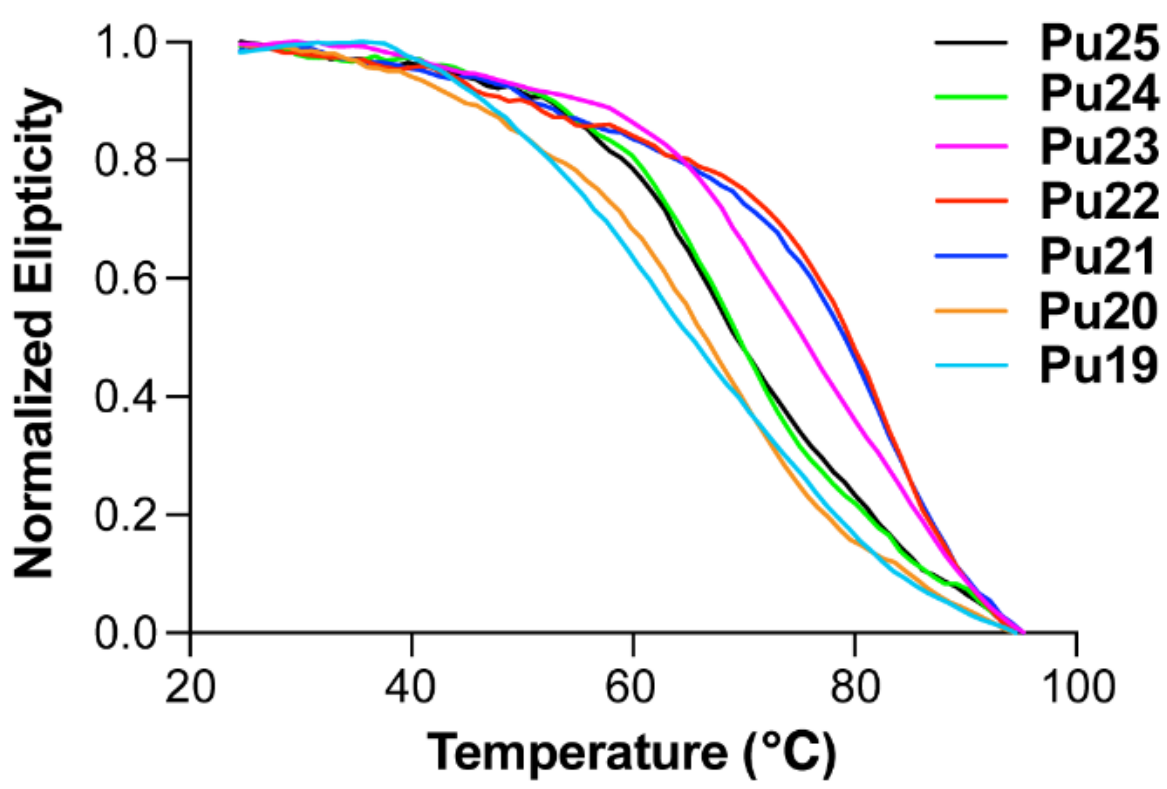

Figure S7. Top: Sequences and melting temperatures of Pu25, Pu24, Pu23, Pu22, Pu21, Pu20, and Pu19 sequences. Middle: CD spectra at 25 °C Bottom: CD melting curves. Sample condition: 5 μM DNA, 100 mM K^+, pH 7.

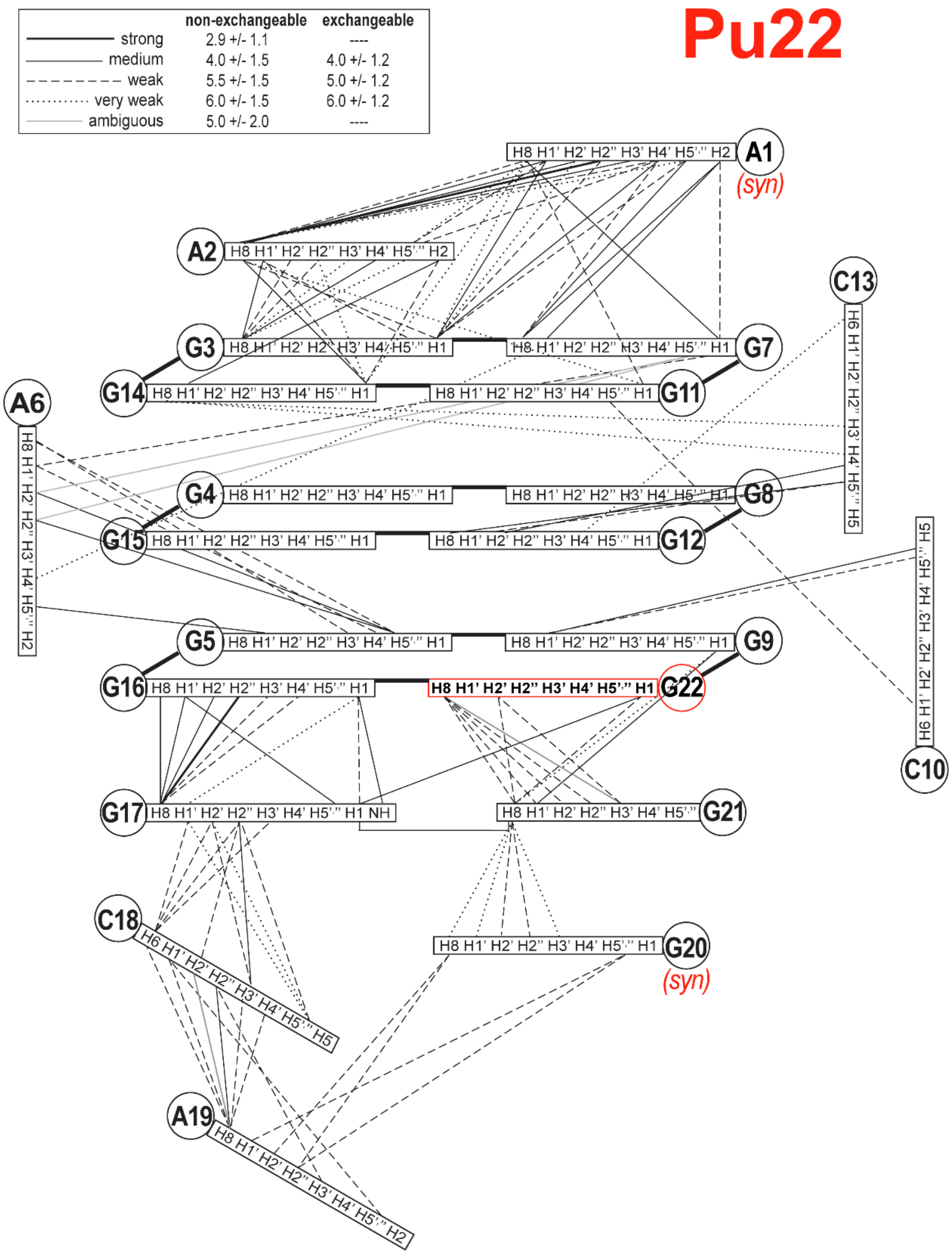

Figure S8. Schematic summary of the capping and loop inter-residue NOEs of the major PDGFR-β Pu22 G-quadruplex.

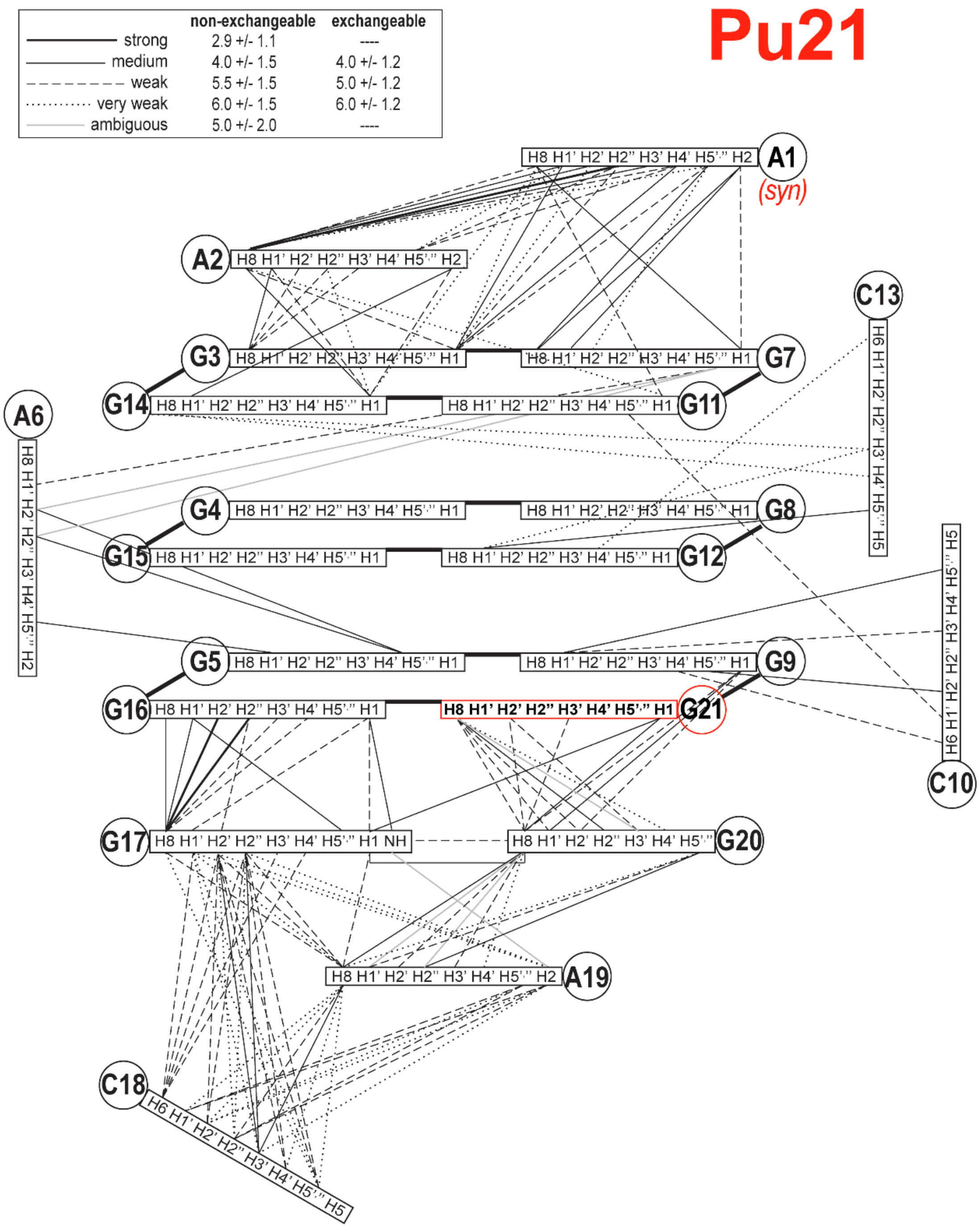

Figure S9. Schematic summary of the capping and loop inter-residue NOEs of the major PDGFR-β Pu21 G-quadruplex.

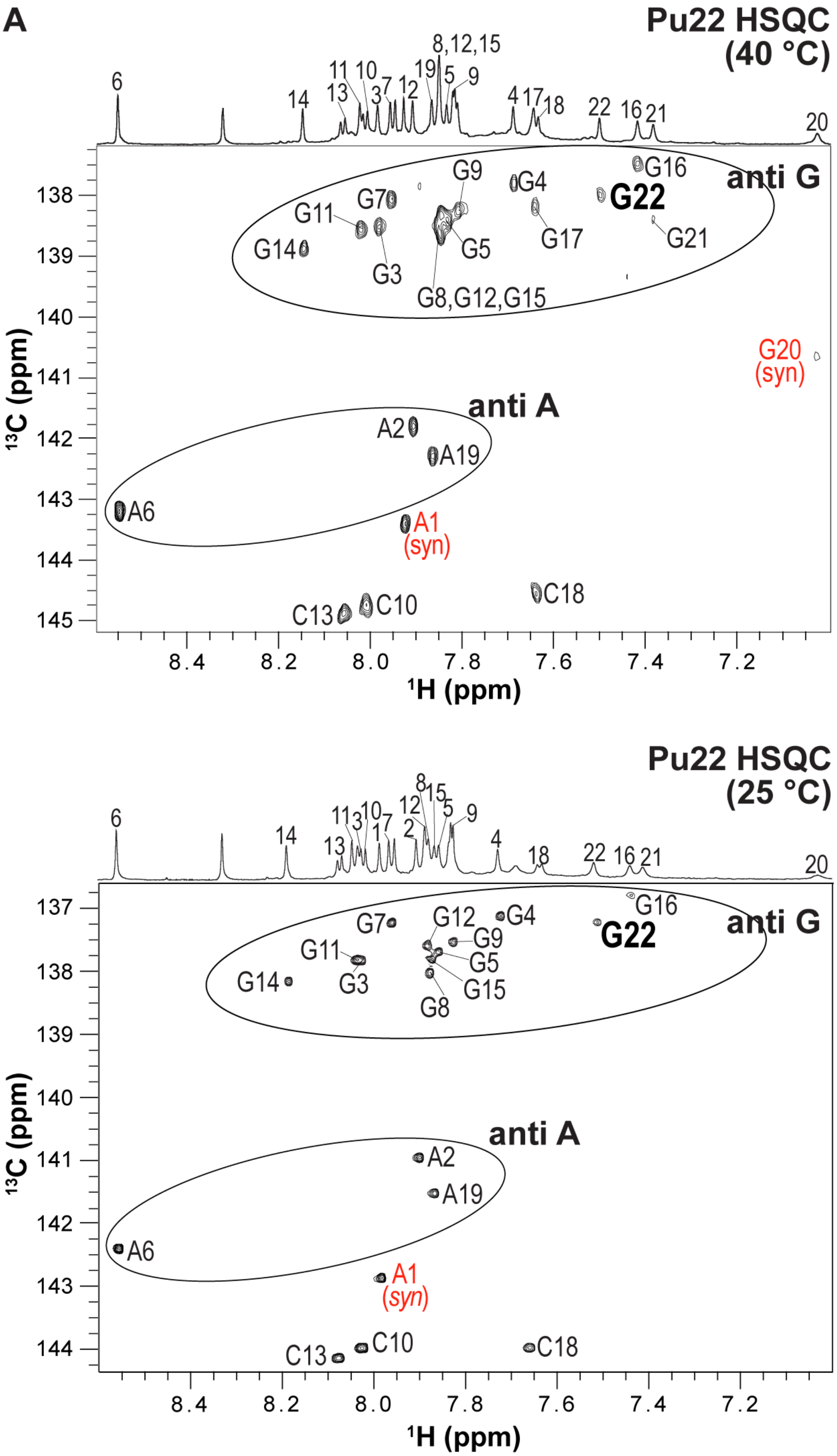
A
Pu22 HSQC
(40 °C)
anti G
G22
G20
(syn)
anti A
A1
(syn)
^{13}C (ppm)
^{1}H (ppm)
Pu22 HSQC
(25 °C)
anti G
G22
anti A
A1
(syn)
^{13}C (ppm)
^{1}H (ppm)

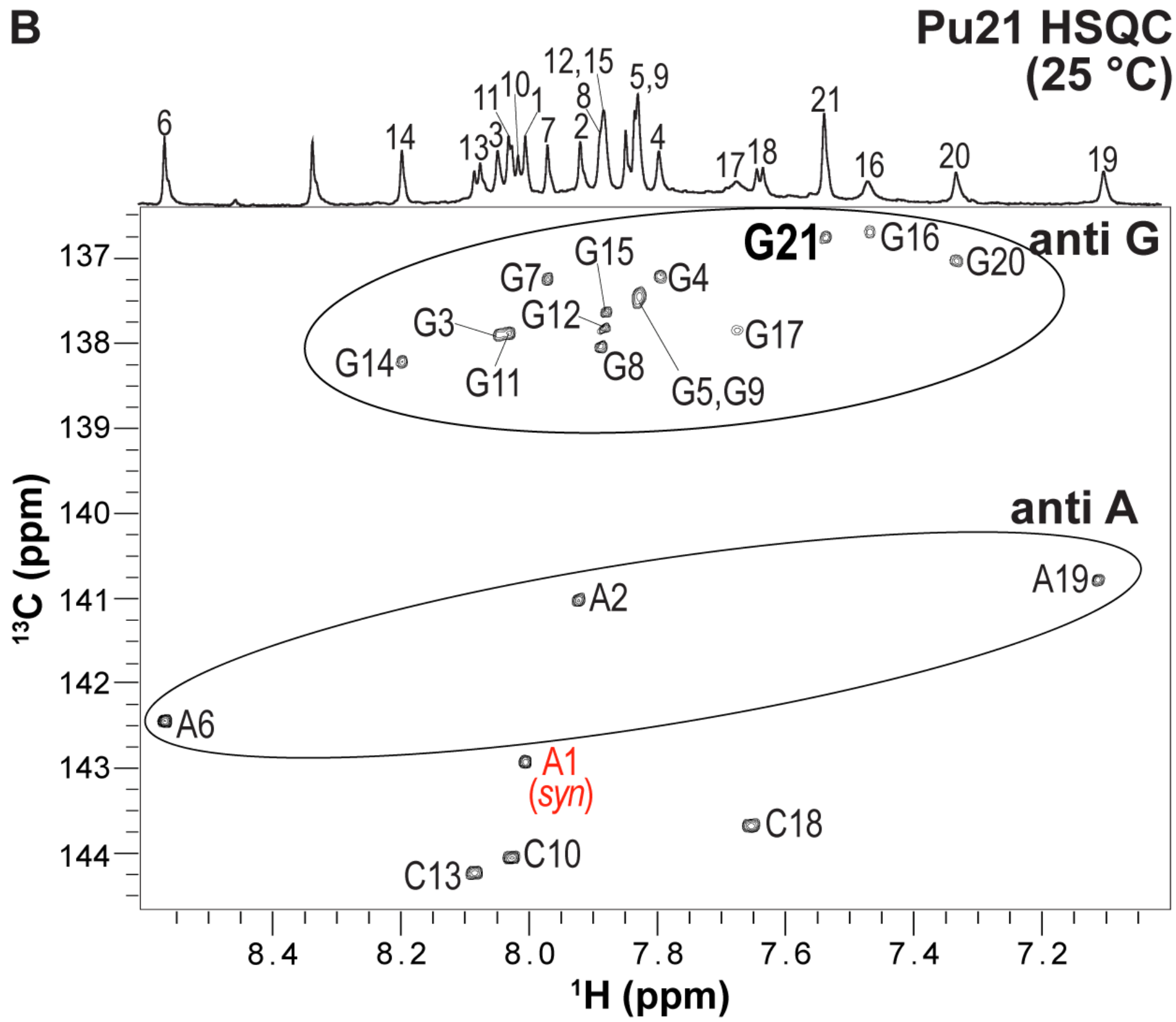

Figure S10. 2D ^{1}H-^{13}C HSQC aromatic spectral regions of the major A) PDGFR-β Pu22 (40, 25 °C) and B) Pu21 (25 °C) G-quadruplexes. Chemical shift regions of A and G *anti* conformers are circled. Bold = Fill-in G22/21 of the Pu22/21 is within *anti* region. Red label = Pu22 G20 and Pu22/21 A1 residues are within *syn* region. NMR sample: Pu22 (0.73 mM), Pu21 (1 mM), 100 mM K^{+}, pH 7, 10 % D_2O/90 % H_2O.

Pu22 NMR Ensemble

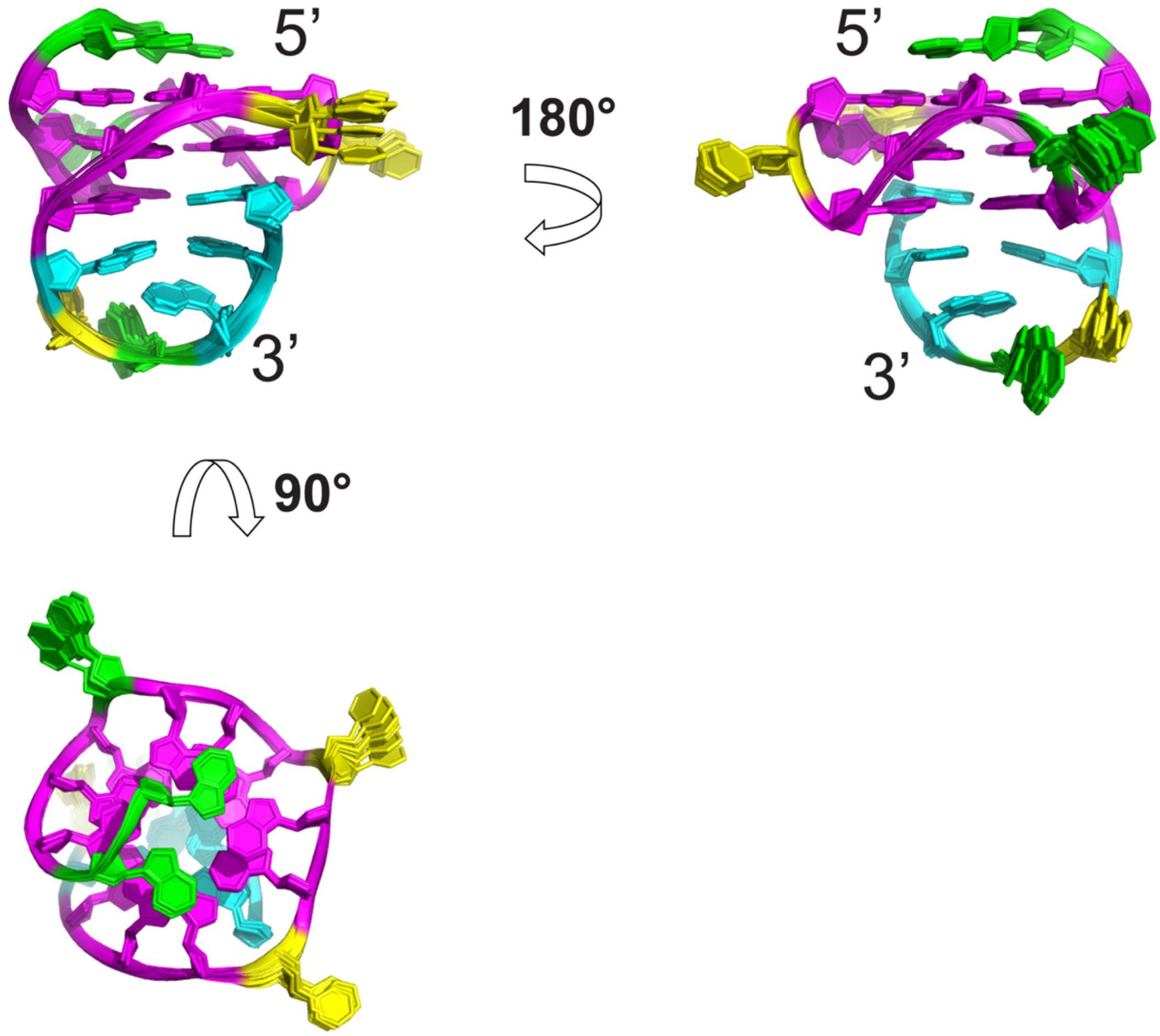

Figure S11. Superposition of the 10 lowest energy structures of the major PDGFR-β Pu22 G-quadruplex.

Pu21 NMR Ensemble

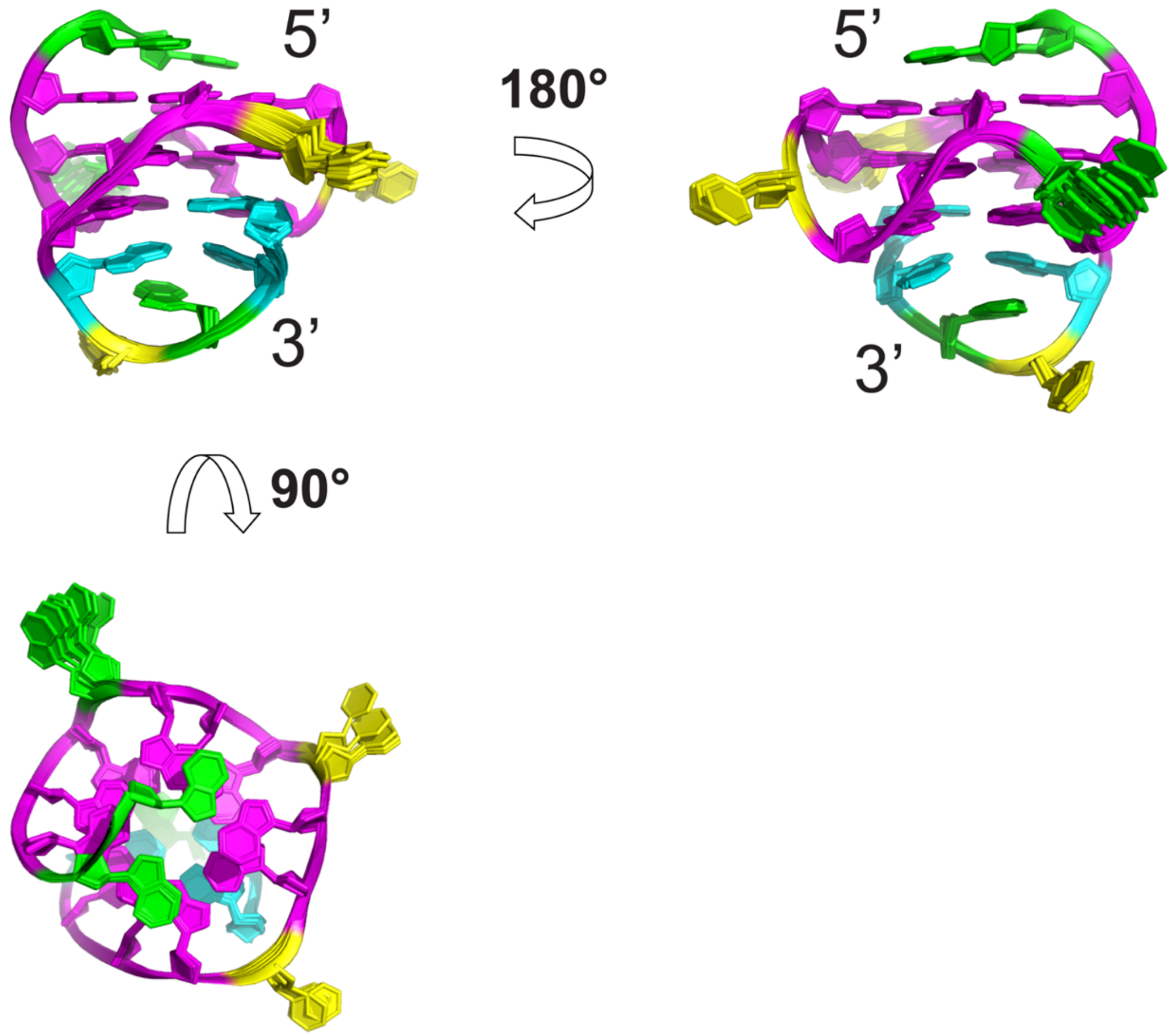

Figure S12. Superposition of the 10 lowest energy structures of the major PDGFR-β Pu21 G-quadruplex.

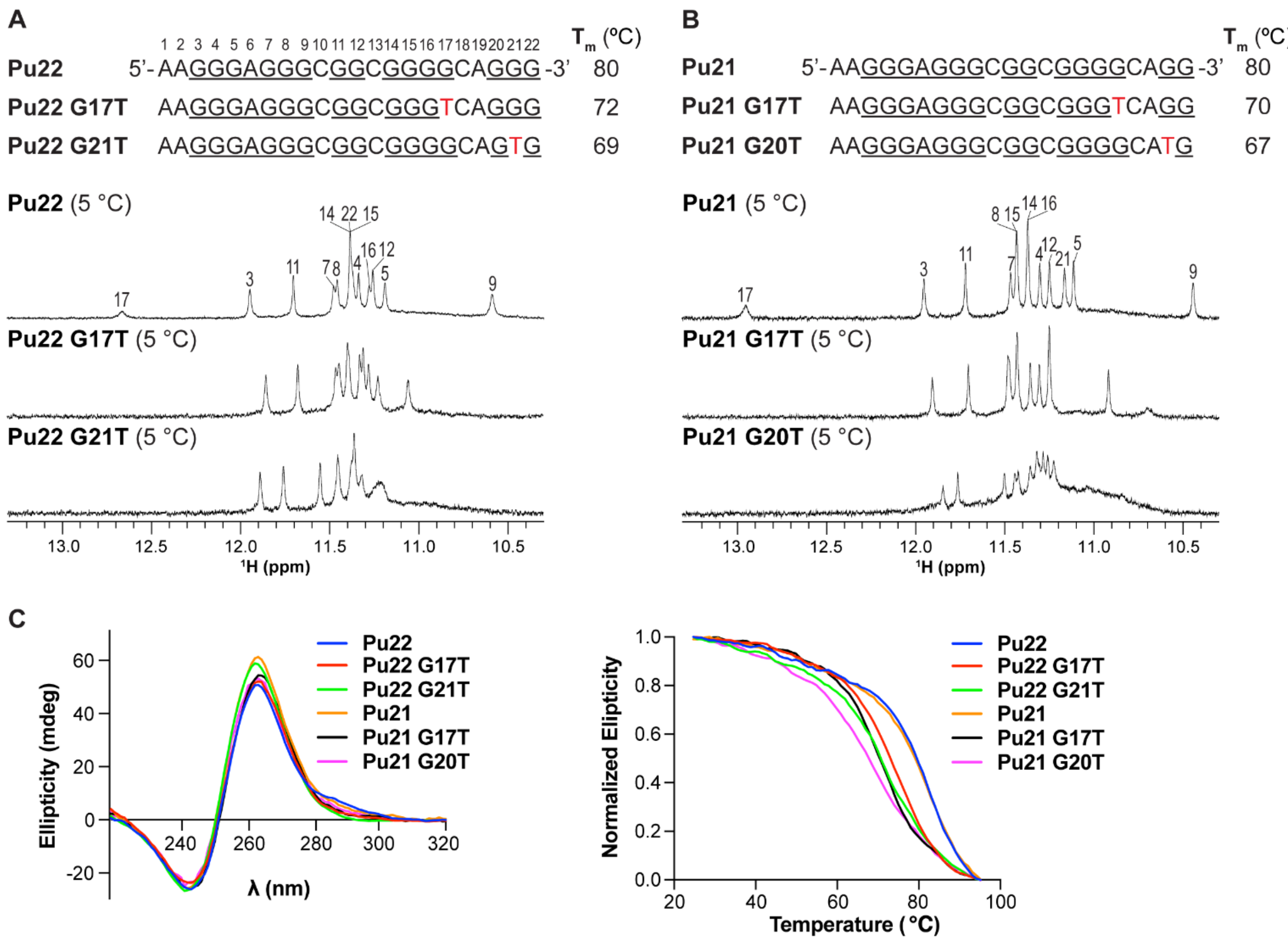

Figure S13. Sequences, CD melting temperatures, and 1D ^{1}H NMR spectra showing the imino proton region for wild-type and corresponding G:G base pair mutant sequences of the major PDGFR-β A) Pu22 and B) Pu21 G-quadruplexes. C) CD spectra (left) and melting curves (right) of all sequences. NMR sample: 150 μM DNA, 100 mM K^+, pH 7, 5 °C, 10 % D_2O/90 % H_2O. CD sample: 5 μM DNA, 100 mM K^+, pH 7, 25 °C.

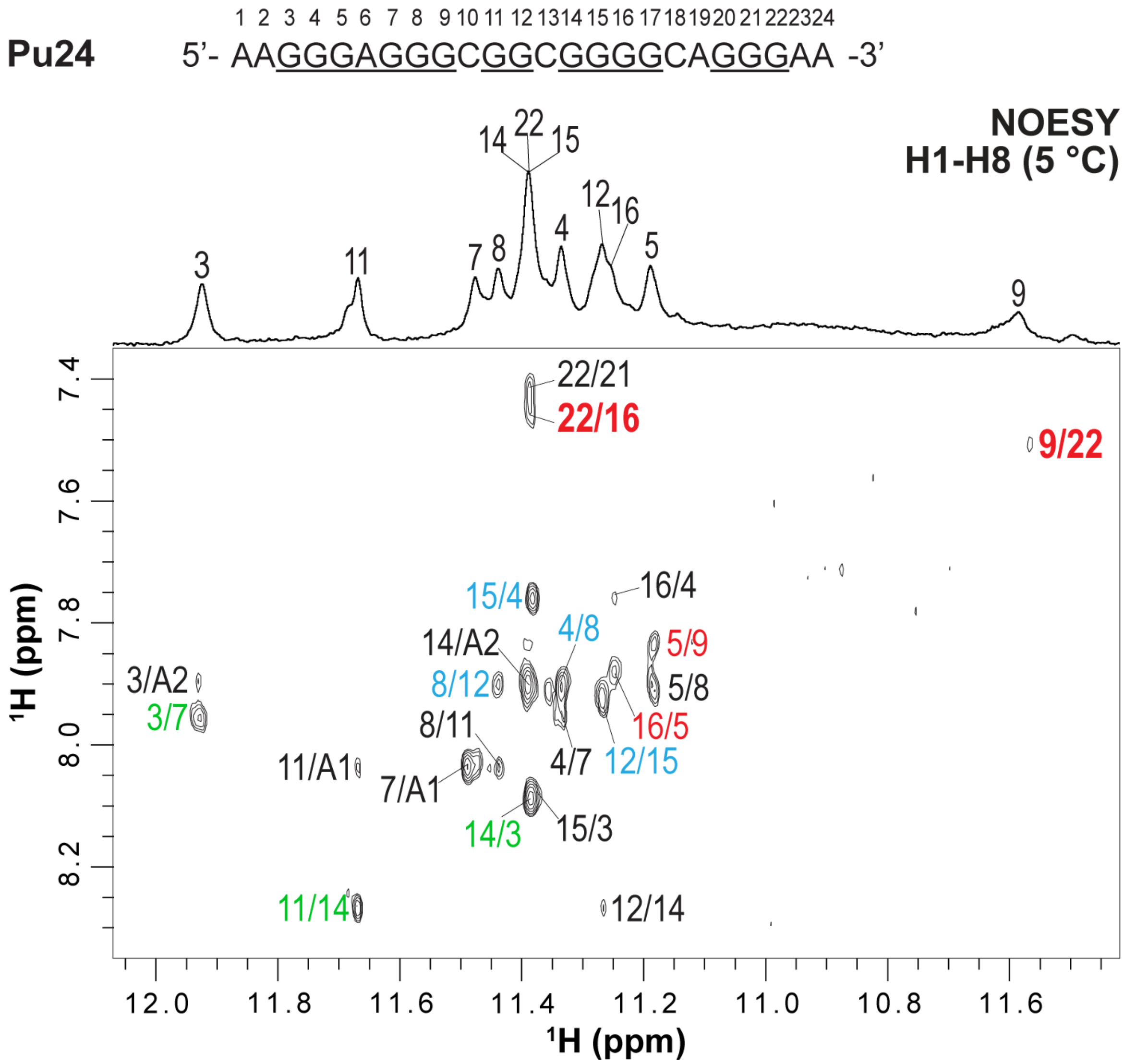

Figure S14. 2D NOESY H1-H8 spectral region of the extended PDGFR-β Pu24 G-quadruplex. Red label = intra-tetrad NOEs defining the G22 filled-in 3′-. Cyan label = intra-tetrad NOEs of the middle tetrad. Green label = intra-tetrad NOEs of the 5′-tetrad. NOESY was measured with a mixing time of 250 ms. Sample condition: 1 mM DNA, 100 mM K^+, pH 7, 5 °C, 10 % D_2O/90 % H_2O.

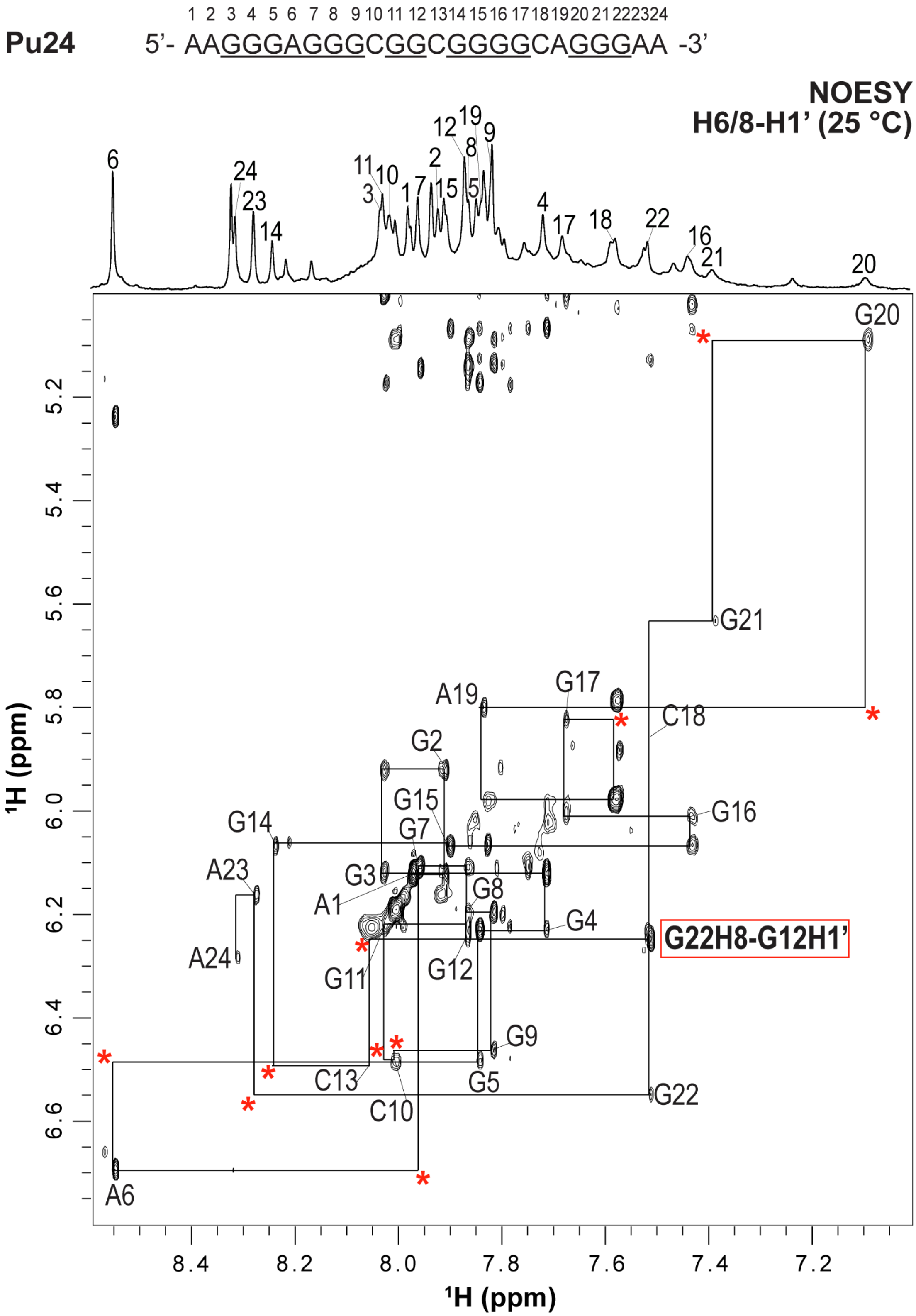

Figure S15. Top: 1D ^{1}H NMR spectrum showing the aromatic region of the extended PDGFR-β Pu24 sequence at with H6/H8 assignments. Bottom: 2D NOESY H6/8-H1′ spectral region of the Pu24 G-quadruplex. Sequential NOE contacts are traced by solid lines. Red box = G22H8-G12H1′ NOE defines the G22 fill-in. Missing NOE cross peaks are marked with an asterisk. NOESY was measured a mixing time of 250 ms. Sample condition: 1 mM DNA, 100 mM K^+, pH 7, 25 °C, 10 % D_2O/90 % H_2O.

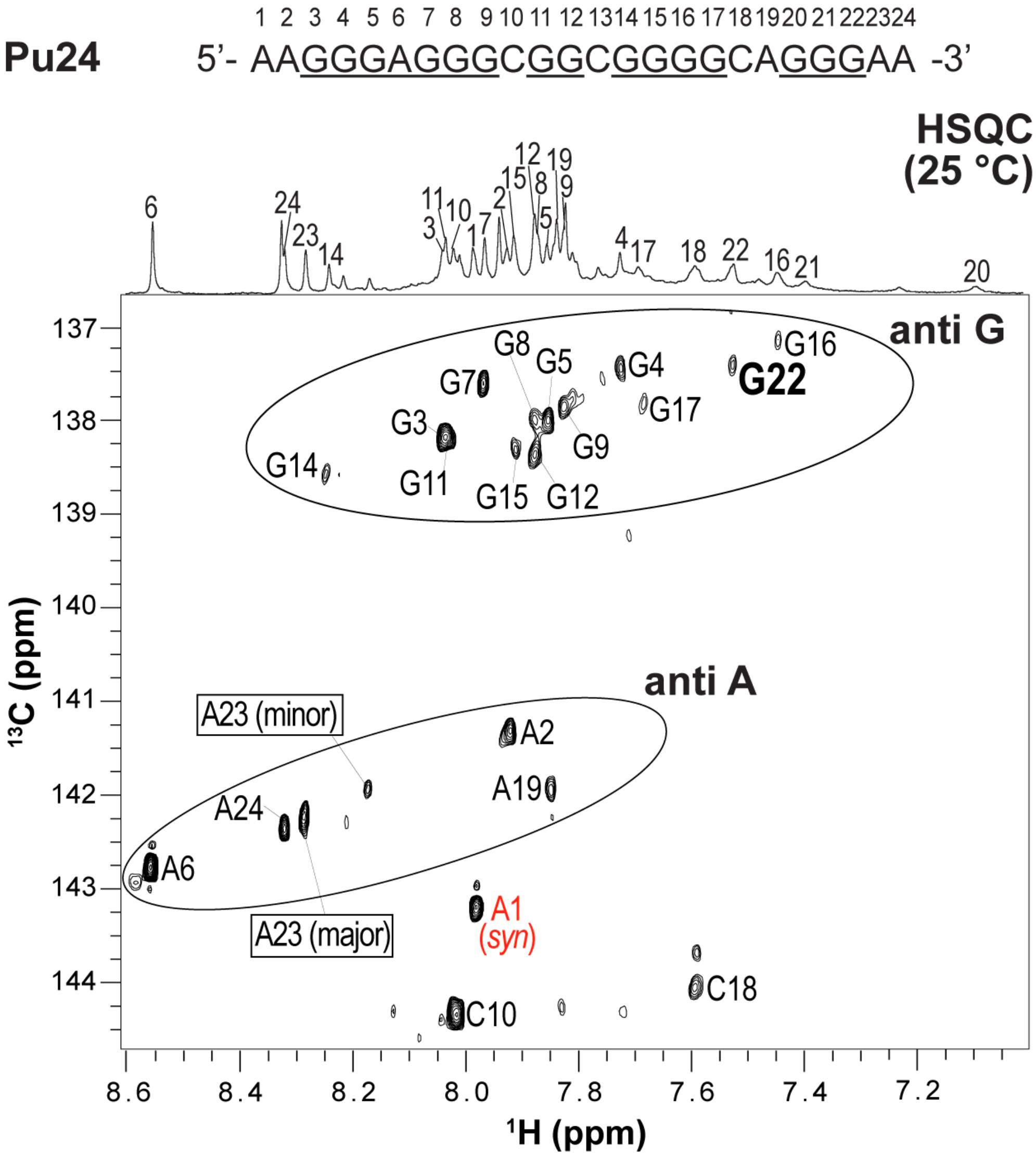

Figure S16. 2D ^{1}H-^{13}C HSQC aromatic spectral region of the extended Pu24 G-quadruplex. Chemical shift regions of A and G *anti* conformers are circled. Bold = Fill-in G22 is within *anti* region. Red label = A1 is within *syn* region. Black box = A23 has two peaks corresponding to the major G22-in and minor G21-in species. NMR sample: 1 mM DNA, 100 mM K^+, pH 7, 25 °C, 10 % D_2O/90 % H_2O.

Pu24 5'- AAGGGAGGGCGGCGGGGCAGGGAA -3'

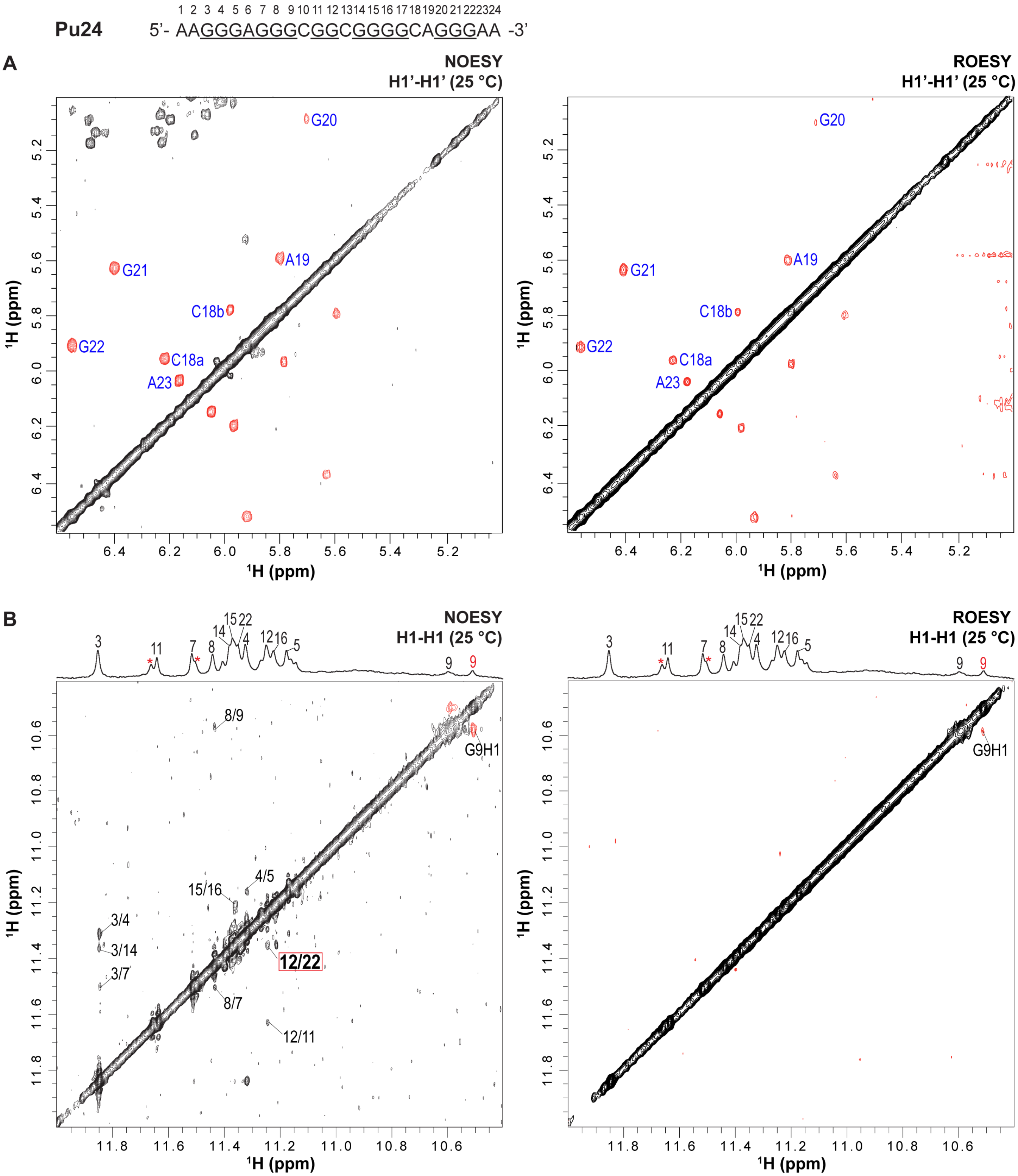

C

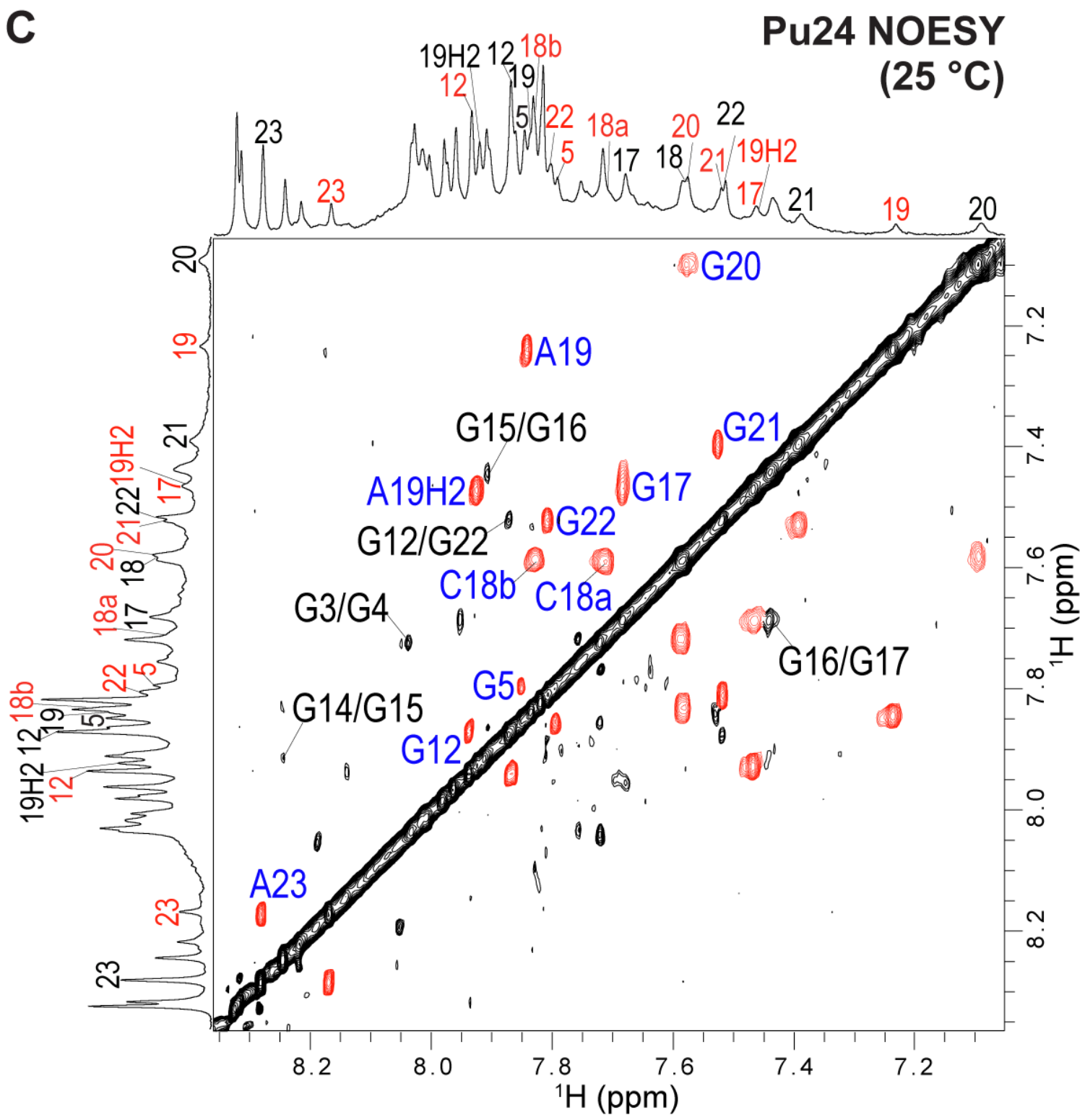

Figure S17. 2D NOESY and 2D ROESY spectra for Pu24 at 25 °C: A) sugar H1′ region, B) aromatic imino H1 region, and C) aromatic H8 region including 1D spectra with assignment for the major (black) and minor (red) species. The exchange cross peaks are in red, whereas NOE cross peaks are in black. (note: peak color does not represent actual phase). Exchange cross peaks involving the 3′ snapback loop are labelled in blue. The mixing time for ROESY is 80 ms, while the mixing time for NOESY is 250 ms. Sample conditions: 1 mM DNA, 100 mM K^+, pH 7, 10 % D_2O/90 % H_2O.

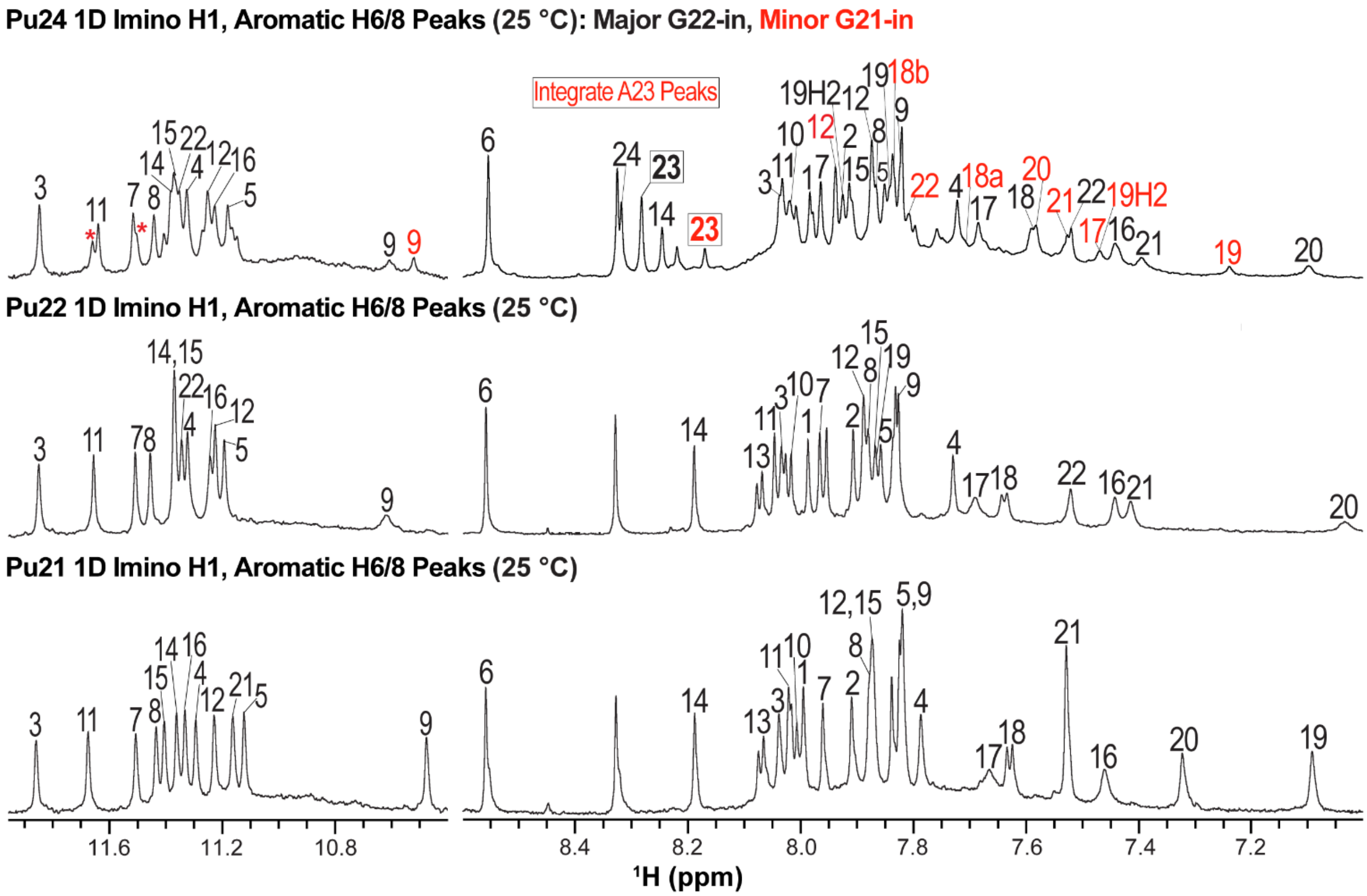

Figure S18. 1D ^{1}H NMR spectra showing imino and aromatic regions of Pu24, Pu22, and Pu21 with assignments. Peaks of the extended Pu24 sequence are labelled in black(major G22-in species), red (minor G21-in species), and with asterisks (unassigned Pu24 minor species). A23H8 peaks of major and minor were integrated to determine the major and minor population as 75 % and 25 %, respectively. NMR sample: 1 mM DNA, 100 mM K^+, pH 7, 25 °C, 10 % D_2O/90 % H_2O.

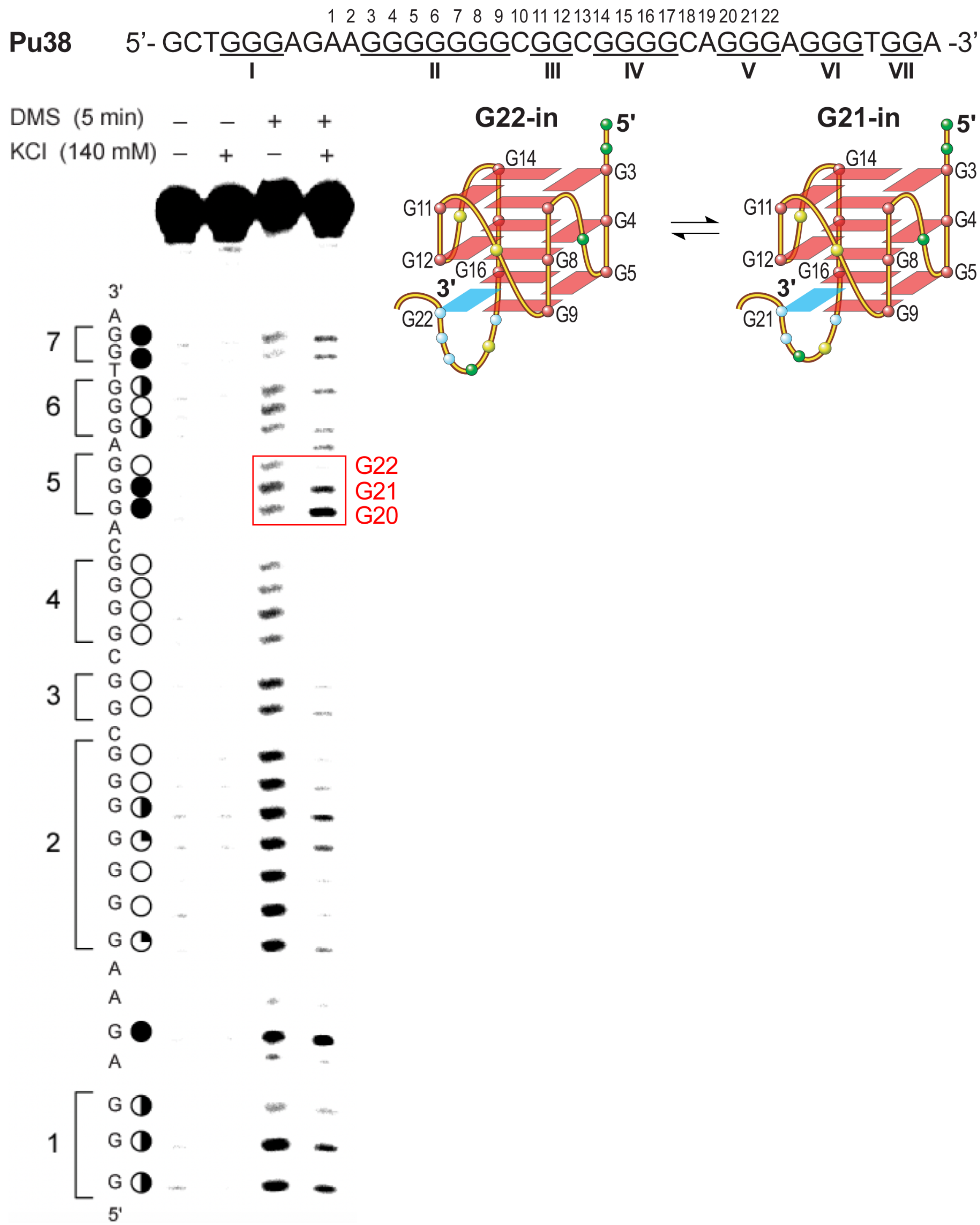

Figure S19. DMS-footprinting data of the full-length PDGFR-β NHE Pu38 sequence (top) in 140 mM K^+, pH 7.6. Our model of equilibrating G22-in and G21-in structures with a snapback-loop is shown. The cleavage at G22, G21, and G20 is labeled with a red box. G20 is fully cleaved consistent with its location in the loop. In contrast, G22 is clearly protected from DMS access indicating its involvement in the G-tetrad core as the major conformation, while G21 shows partial protection as a minor conformation. Modified with permission from Chen, Y.; Agrawal, P.; Brown, R. V.; Hatzakis, E.; Hurley, L.; Yang, D. The Major G-Quadruplex Formed in the Human Platelet-Derived Growth Factor Receptor β Promoter Adopts a Novel Broken-Strand Structure in K^+ Solution. J. Am. Chem. Soc. 2012, 134, 13220–13223. DOI: 10.1021/ja305764d. Copyright 2026 American Chemical Society.

Table S1. Names, sequences, and CD melting temperatures (T_m) of oligonucleotides used in this study. G-core guanines are shown in bold and mutations are shown in red.

**Name**	**Sequence** (5'-3')	T_m (°C)[a]
**Pu19 vG4**	AA **GGG** A **GGG** C **GG** C **GGG** GCA	66
**Pu20**	AA **GGG** A **GGG** C **GG** C **GGG** GCAG	67
**Pu21**	AA **GGG** A **GGG** C **GG** C **GGG** GCAG**G**	80
**Pu21 G17T**	AA **GGG** A **GGG** C **GG** C **GGG** TCAG**G**	70
**Pu21 G20T**	AA **GGG** A **GGG** C **GG** C **GGG** GCAT**G**	67
**Pu22**	AA **GGG** A **GGG** C **GG** C **GGG** GCAGG **G**	80
**Pu22 G17T**	AA **GGG** A **GGG** C **GG** C **GGG** TCAGG **G**	72
**Pu22 G21T**	AA **GGG** A **GGG** C **GG** C **GGG** GCAGT **G**	69
**Pu23**	AA **GGG** A **GGG** C **GG** C **GGG** GCAGG G A	75
**Pu24**	AA **GGG** A **GGG** C **GG** C **GGG** GCAGG G AA	69
**Pu25**	AA **GGG** A **GGG** C **GG** C **GGG** GCAGG G AAA	69

[a] Measured with 5 μM DNA, 100 mM K^+, pH 7.

Table S2. ^{1}H chemical shifts (ppm) of the major PDGFR-β Pu22 G-quadruplex.[a]

	H1'	H2'	H2"	H3'	H4'	H5'/H5"[b]	imino	H2/H5	H8/H6
**A1**	6.12	2.34	2.42	4.68	4.03	3.55/3.55	---	7.82	7.98
**A2**	5.91	2.25	2.59	4.84	4.15	3.55/3.87	---	7.82	7.90
**G3**	6.12	2.81	3.04	5.00	4.49	4.02/4.15	11.84	---	8.03
**G4**	6.22	2.68	2.99	5.06	4.59	4.33/4.33	11.31	---	7.72
**G5**	6.48	2.65	2.82	5.17	4.67	4.37/4.43	11.18	---	7.86
**A6**	6.69	2.93	2.93	5.24	4.72	4.33/4.67	---	8.32	8.56
**G7**	6.10	2.44	2.93	5.14	4.51	4.31/4.41	11.50	---	7.96
**G8**	6.19	2.65	2.96	5.09	4.40	4.30/4.30	11.45	---	7.88
**G9**	6.46	2.65	2.77	5.13	4.67	4.32/4.40	10.60	---	7.83
**C10**	6.48	2.44	2.79	5.09	4.54	4.33/4.42	---	6.19	8.02
**G11**	6.23	2.44	2.97	5.19	4.52	4.22/4.36	11.65	---	8.04
**G12**	6.26	2.68	2.91	5.18	4.66	4.22/4.37	11.22	---	7.88
**C13**	6.50	2.38	2.78	5.13	4.62	4.38/4.53	---	6.21	8.08
**G14**	6.02	2.55	2.82	4.88	4.52	4.29/4.40	11.36	---	8.19
**G15**	6.07	2.63	2.86	5.05	4.58	4.21/4.26	11.37	---	7.87
**G16**	6.02	2.41	2.86	5.01	4.50	4.17/4.33	11.24	---	7.44
**G17**	5.82	2.48	2.57	4.99	4.30	4.20/4.20	---	---	7.68
**C18**	5.99	1.79	2.25	4.70	4.20	3.97/4.07	---	6.01	7.66
**A19**	5.85	1.75	1.93	4.41	3.96	3.74/3.78	---	7.95	7.87
**G20**	5.07	1.85	2.24	4.71	4.29	3.57/3.60	---	---	7.04
**G21**	5.65	2.09	2.09	4.52	4.15	3.10/3.85	---	---	7.41
**G22**	6.45	2.47	2.78	4.61	4.51	4.15/4.37	11.33	---	7.51

[a] Measured at 25 °C with 1.6 mM DNA, 10 mM K^+, pH 7, 10 % D_2O/90 % H_2O.
[b] No stereospecific assignment.

Table S3. ^{13}C chemical shifts (ppm) of the major PDGFR-β Pu22 G-quadruplex.[a]

	C1'	C2'	C3'	C4'	C5'	C8/6	C2/5
**A1**	88.42	40.85	79.17	89.84	64.54	142.9	154.26
**A2**	87.29	42.44	---	86.69	68.1	141	154.57
**G3**	84.45	39.93	79.73	88.33	69.75	137.87	---
**G4**	84.52	---	79.26	87.2	68.05	137.19	---
**G5**	85.17	41.49	81.16	85.67	69.06	137.72	---
**A6**	86.49	42.31	79.31	87.65	68.71	142.46	155.48
**G7**	87.13	43.96	79.13	87.33	68.05	137.27	---
**G8**	84.34	43	79.33	---	68.39	138.07	---
**G9**	84.92	41.24	81.3	85.42	69.19	137.61	---
**C10**	88.54	---	77.79	87.23	67.5	144.03	99.42
**G11**	86.62	43.98	81.09	87.9	69.23	137.87	---
**G12**	84.18	41.47	79.45	86.81	68.75	137.64	---
**C13**	89.23	42.12	79.87	88.29	68.7	144.18	99.46
**G14**	88.3	43.11	---	87.04	68.76	138.22	---
**G15**	84.8	43.09	80.89	87.67	68.81	137.84	---
**G16**	84.52	40.28	80.63	87.45	69.38	136.85	---
**G17**	83.65	41.14	76.97	86.33	66.88	137.49	---
**C18**	88.18	40.21	79.07	86.87	68.01	144.02	99.15
**A19**	85.43	39.68	78.77	87.56	67.69	141.58	155.19
**G20**	86.3	37.56	---	86.26	67.1	---	---
**G21**	84.67	---	77.81	87.68	67.57	---	---
**G22**	90.06	44.73	74.37	89.65	---	137.28	---

[a] Measured at 25 °C with 1.6 mM DNA, 10 mM K^+, pH 7, 10 % D_2O/90 % H_2O.

Table S4. ^{1}H chemical shifts (ppm) of the major PDGFR-β Pu21 G-quadruplex.[a]

	H1'	H2'	H2"	H3'	H4'	H5'/H5"[b]	imino	H2/H5	H8/H6
**A1**	6.13	2.36	2.44	4.69	4.04	3.5	---	7.83	8.00
**A2**	5.92	2.26	2.60	4.85	4.14	3.55/3.87	---	7.84	7.92
**G3**	6.11	2.81	3.06	5.00	4.49	4.15/4.03	11.85	---	8.04
**G4**	6.22	2.73	3.01	5.08	4.60	4.36/4.32	11.29	---	7.79
**G5**	6.48	2.64	2.78	5.18	4.66	4.37/4.43	11.11	---	7.82
**A6**	6.69	2.94	2.94	5.24	4.73	4.33	---	8.33	8.56
**G7**	6.10	2.46	2.92	5.13	4.51	4.32/4.41	11.50	---	7.96
**G8**	6.20	2.66	2.96	5.09	4.59	4.30	11.43	---	7.88
**G9**	6.42	2.64	2.78	5.14	4.66	4.37/4.32	10.46	---	7.82
**C10**	6.48	2.45	2.78	5.08	4.52	4.31/4.41	---	6.20	8.02
**G11**	6.22	2.41	2.95	5.18	4.52	4.22/4.36	11.67	---	8.02
**G12**	6.23	2.65	2.92	5.17	4.65	4.22/4.36	11.22	---	7.87
**C13**	6.50	2.38	2.79	5.12	4.63	4.53/4.37	---	6.22	8.08
**G14**	6.04	2.59	2.82	4.89	4.55	4.41/4.29	11.35	---	8.19
**G15**	6.07	2.67	2.80	5.07	4.59	4.23/4.28	11.39	---	7.88
**G16**	6.06	2.48	2.86	5.02	4.51	4.34/4.16	11.32	---	7.46
**G17**	5.84	2.53	2.53	4.96	4.29	4.29/4.20	12.85	---	7.67
**C18**	5.92	1.62	1.98	4.41	4.12	3.96	---	6.00	7.65
**A19**	5.61	1.79	2.02	4.47	3.98	3.41/3.50	---	7.54	7.11
**G20**	5.62	1.21	2.01	4.54	4.16	3.79/3.90	---	---	7.33
**G21**	6.44	2.59	2.77	4.58	4.47	4.53/4.21	11.15	---	7.53

[a] Measured at 25 °C with 1 mM DNA, 100 mM K^+, pH 7, 10 % D_2O/90 % H_2O.
[b] No stereospecific assignment.

Table S5. ^{13}C chemical shifts (ppm) of the major PDGFR-β Pu21 G-quadruplex.[a]

	**C1'**	**C2'**	**C3'**	**C4'**	**C5'**	**C8/6**	**C2/5**
**A1**	88.37	40.82	79.20	89.85	64.54	142.94	154.22
**A2**	87.28	42.39	---	86.71	68.10	141.04	154.60
**G3**	84.43	39.92	79.78	88.43	69.75	137.93	---
**G4**	84.41	42.50	79.31	87.20	68.76	137.25	---
**G5**	85.04	41.59	81.01	85.60	68.99	137.50	---
**A6**	86.51	42.29	79.32	87.60	68.07	142.46	155.47
**G7**	87.21	43.91	78.85	87.22	67.96	137.28	---
**G8**	84.47	43.11	79.48	87.18	68.43	138.07	---
**G9**	84.85	41.00	81.30	85.35	69.23	137.48	---
**C10**	88.47	42.13	77.60	87.88	67.39	144.06	99.37
**G11**	86.59	44.08	81.01	87.18	68.70	137.92	---
**G12**	84.21	41.47	79.43	86.81	69.25	137.66	---
**C13**	89.23	42.08	79.83	88.29	68.70	144.23	99.43
**G14**	88.39	43.25	77.54	87.17	68.93	138.25	---
**G15**	84.78	43.09	80.80	87.52	68.92	137.86	---
**G16**	84.49	40.21	80.20	87.38	69.37	136.73	---
**G17**	83.56	41.12	76.67	85.89	66.89	137.87	---
**C18**	87.99	40.47	78.04	86.90	67.35	143.68	99.26
**A19**	85.97	38.52	79.35	87.35	67.58	140.82	154.48
**G20**	84.16	38.19	77.72	87.30	67.74	137.05	---
**G21**	89.75	44.27	73.34	89.29	66.63	136.78	---

[a] Measured at 25 °C with 1 mM DNA, 100 mM K^+, pH 7, 10 % D_2O/90 % H_2O.

Table S6. Sequential and long-range intramolecular NOE interactions of the 5′-capping residues A1-A2 for Pu22.[a]

A1		H1'	H2'	H2"	H3'	H4'	H5'	H5"	H2	H8
**A2**	**H5'**	w				w				
	**H5"**	w								
	**H8**	m	m	s	m	w	vw			w
**G3**	**H1**	m	vw	w		m	w	w		vw
**G7**	**H1'**								m	
	**H1**								w	m
	**H8**					w	m		m	
**G11**	**H1**									w
**G14**	**H1**									vw

A2		H1'	H2'	H2"	H3'	H4′	H5'	H5"	H2	H8
**G3**	**H5'**	w								
	**H1**									w
	**H8**	m	w	w	vw	m	vw	vw		
**G11**	**H1**									vw
**G14**	**H1'**								m	
	**H1**	w		vw					w	m

[a]Cross peak intensities are classified as strong (s), medium (m), weak (w), or very weak (v).

Table S7. Sequential and long-range intramolecular NOE interactions of the 3′-capping residues G17-G21 for Pu22.[a]

G17		H1'	H2'	H2''	H3'	H5'	H5''	H1	H21	H8
**G16**	**H1'**					m	m			m
	**H2'**									m
	**H2''**									s
	**H3'**									w
	**H4'**									w
	**H1**	vw						w	m	
	**H8**									m
**C18**	**H2'**			w						
	**H3'**		w	m						
	**H5'**	vw	vw	w						
	**H5''**	vw	vw	w						
	**H6**	w	w	w	w					
**G21**	**H8**							m		
**G22**	**H1**							m		

C18		H1'	H2'	H2''	H3'	H4'	H6
**A19**	**H3'**			w			
	**H5'**	w					
	**H5''**	w					
	**H8**	w	amb	m	w	w	w

A19		H1'	H2'	H2''
**G20**	**H5'**	w		w
	**H5''**	w		w
	**H8**		w	w

G20		H1'	H2'	H2''	H3'	H8
**G21**	**H8**	vw	w	w	vw	vw

G21		H1'	H2'	H2''	H3'	H8
**G22**	**H2'**				w	w
	**H1**					w
	**H8**	w	w	w	amb	w
**G9**	**H1**	m				vw

[a]Cross peak intensities are classified as strong (s), medium (m), weak (w), very weak (v), or ambiguous (a) in case of overlapped resonances.

Table S8. Sequential and long-range intramolecular NOE interactions of the 5′-capping residues A1-A2 for Pu21.[a]

A1		H1'	H2'	H2''	H3'	H4'	H5'	H5''	H2	H8
**A2**	**H5'**	vw		w		w				
	**H5''**	vw								
	**H8**	m	m	s	m	w	vw	vw		w
**G3**	**H1**	m	vw	w		m	w	w		vw
**G7**	**H1'**								m	
	**H2'**							vw		
	**H1**								w	m
	**H8**						m		m	
**G11**	**H1**									w
**G14**	**H1**									vw

A2		H1'	H2'	H2''	H3'	H2	H8
**G3**	**H1**						w
	**H8**	m	w	w	w		
**G11**	**H1**						vw
**G14**	**H1'**					m	
	**H1**	w		vw		w	m

[a]Cross peak intensities are classified as strong (s), medium (m), weak (w), or very weak (v).

Table S9. Sequential and long-range intramolecular NOE interactions of the 3′-capping residues G17-G20 for Pu21.[a]

G17		H1'	H2'	H2''	H3'	H4'	H5'	H5''	H1	H21	H8
**G16**	**H1'**						m	m			m
	**H2'**										s
	**H2''**		w								s
	**H3'**										w
	**H4'**										w
	**H1**	w							w	m	
	**H8**										m
**C18**	**H2'**		w	w							
	**H2''**		vw	vw							
	**H3'**	vw	m	m							w
	**H4'**		w	w							
	**H5'**	vw	w	w							vw
	**H5''**	vw	w	w							vw
	**H6**	w	w	w	w	w					
**A19**	**H8**	vw	w	w					w		w
	**H2**	vw	vw	vw						amb	
**G20**	**H8**								m	w	
**G21**	**H1**								m		

C18		H1'	H2'	H2''	H3'	H4'	H5'	H5''
**A19**	**H5'**	w	vw	w				
	**H5''**	w	vw	w				
	**H2**	w	vw	w	vw			
	**H8**	vw	vw	w	m	vw	vw	vw

A19		H1'	H2'	H2''	H3'	H4'	H8
**G20**	**H5'**	w		m			vw
	**H5''**	w		m			vw
	**H8**	amb	w	amb	w	vw	m

G20		H1'	H2'	H2''	H3'	H4'	H8
**G21**	**H2'**				w		w
	**H3'**						w
	**H1**						m
	**H8**	w	w	m	amb	vw	w
**G9**	**H1**	m	w				w

[a]Cross peak intensities are classified as strong (s), medium (m), weak (w), very weak (v), or ambiguous (a) in case of overlapped resonances.

Table S10. NMR restraints and structural statistics of the Pu22 and Pu21 G-quadruplex NMR structures.

**Calculation Restraints**	**Pu22**	**Pu21**
**NOE Restraints**		
intra-residue	484	428
sequential	183	181
long-range	88	101
**Other Restraints**		
hydrogen bond	52	52
dihedral angle	22	21
planarity	36	36
**Structural Statistics**		
**Pairwise Heavy Atom RMSD (Å)**		
G-tetrad core	0.51	0.52
all residues	0.71	0.61
**Violations**		
mean NOE violation (Å)	0.05	0.05
maximum NOE violation (Å)	0.11	0.15
